# Associations of Frailty Index and Composite Dietary Antioxidant Index With Major Ocular Diseases and Analysis of Component Contributions: A Cross-Sectional Study

**DOI:** 10.1167/tvst.15.4.25

**Published:** 2026-04-27

**Authors:** Huan Ju, Lingyi Jin, Xuefeng Qin, Yue Tian, Hui Peng, Xing Wang

**Affiliations:** 1 Department of Ophthalmology, The First Affiliated Hospital of Chongqing Medical University. Chongqing Key Laboratory for the Prevention and Treatment of Major Blinding Eye Diseases, Chongqing, China; 2 Department of Clinical Medicine, Chongqing Medical University, Chongqing, China

**Keywords:** ocular diseases, composite dietary antioxidant index, frailty index, national health and nutrition examination survey

## Abstract

**Purpose:**

To evaluate the independent and joint effects of the frailty index (FI) and composite dietary antioxidant index (CDAI) with major ocular diseases and quantify contributions of their components.

**Methods:**

We analyzed 4455 U.S. adults aged ≥40 years from NHANES 2005–2008. Frailty was defined using a 49-item deficit accumulation FI (FI > 0.21 considered frail), and CDAI was derived from six antioxidants (carotenoids, vitamins A, C, E, selenium, zinc). Weighted multivariable logistic regression, variable importance, restricted cubic spline analyses, and sensitivity analyses assessed associations with retinopathy, cataract, diabetic retinopathy (DR), glaucoma, and age-related macular degeneration.

**Results:**

Higher FI was associated with higher odds of all major ocular diseases (any ocular disease: odds ratio [OR] = 1.35; DR: OR = 2.21; glaucoma: OR = 1.34), whereas higher CDAI was associated with lower odds (any ocular disease: OR = 0.97; glaucoma: OR = 0.91). Participants with high FI and low CDAI had the highest odds, based on predefined cutoff categories (any ocular disease: OR = 2.22; DR: OR = 4.92; cataract: OR = 2.50; glaucoma: OR = 3.18; all *P* < 0.05). Exploratory analyses showed that CDAI contributions varied by disease, whereas chronic disease burden dominated among FI domains.

**Conclusions:**

Higher FI and lower CDAI were independently and in combination associated with major ocular diseases, highlighting the relevance of considering frailty status and dietary antioxidant profiles together in ocular health.

**Translational Relevance:**

Higher FI and lower CDAI show combined associations with major ocular diseases, emphasizing their relevance to vision-related health.

## Introduction

With global population aging, visual impairment has become a major public health challenge. By 2050, approximately 474 million people are expected to have moderate-to-severe vision loss.^1^ Among older adults, cataracts, glaucoma, age-related macular degeneration (AMD), and diabetic retinopathy (DR) are the leading causes.^2^ Visual impairment reduces daily functioning and social participation, increases risks of falls, depression, and dependency, and imposes substantial public health and socioeconomic burdens.^3^ These trends highlight the need for reliable assessment tools to identify individuals at higher risk of blinding eye diseases.

The eye is highly vulnerable to oxidative stress because of continuous light exposure and high metabolic activity. Disruption of redox homeostasis contributes to various ocular diseases affecting the ocular surface, lens, retina, and optic nerve.^4^ Dietary antioxidants help counter oxidative stress and inflammation,^5^ the composite dietary antioxidant index (CDAI) integrates carotenoids, vitamins E, C, and A, selenium, and zinc to assess overall dietary antioxidant intake.^6^ Prior studies have shown that antioxidants such as vitamin E, zinc, and selenium may slow AMD progression and reduce DR risk.^7^^,^^8^

With population aging, frailty has also emerged as a major public health concern, closely associated with stroke, falls, and all-cause mortality.^9^ The frailty index (FI), a validated indicator of biological aging, quantifies cumulative health deficits across multiple domains. Higher FI values reflect reduced physiological reserve and greater vulnerability to stressors.^10^ Given its high prevalence and predictive value for adverse outcomes, FI is a key component of geriatric risk assessment. Evidence suggests that frailty is associated with visual field loss and impaired near vision.^11^^,^^12^

Accumulating evidence indicates a strong link between frailty and oxidative stress,^13^ which contributes to frailty through cellular damage, redox imbalance, and impaired muscle metabolism.^14^ CDAI and FI reflect oxidative stress and inflammatory burden from complementary perspectives: CDAI represents dietary antioxidant capacity, whereas FI quantifies cumulative physiological deficits. A combined evaluation could provide a more comprehensive picture of oxidative–inflammatory status and reveal shared mechanisms linking nutrition, frailty, and ocular diseases. Although both frailty and CDAI have been individually associated with ocular health, most studies have examined them separately, overlooking potential interactions and shared biological pathways. Few studies have jointly investigated CDAI and FI or quantified the relative contributions of individual antioxidant components and FI domains to ocular disease risk. These gaps limit our understanding of potentially complementary mechanisms connecting antioxidant status and frailty. Therefore this study simultaneously analyzed FI and CDAI to assess their independent and combined associations with major ocular diseases and to determine the relative importance of each antioxidant component and FI domain, providing insights for characterizing associations and understanding potential mechanisms.

## Methods

### Study Population

The data for this study were obtained from the National Health and Nutrition Examination Survey (NHANES), a nationally representative cross-sectional survey of the noninstitutionalized U.S. population conducted biennially since 1999. The NHANES protocol was approved by the NCHS Institutional Review Board (Protocol no. 2005-06), and all participants provided written informed consent. Analyses were performed using de-identified data in accordance with the Declaration of Helsinki; no additional ethical approval was required.

**Figure 1.**
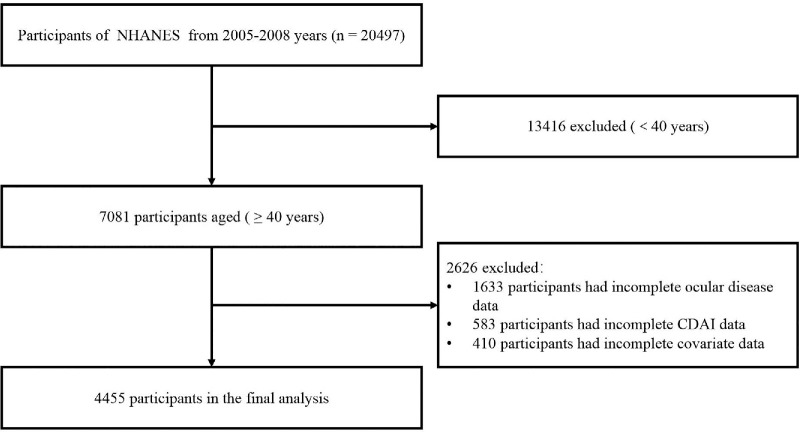
The flowchart of study participants.

The analysis was conducted between September 15 and October 8, 2025, covering the 2005–2008 cycles, which offered the combination of ocular, dietary, and frailty data needed for the present study. A total of 7081 participants aged ≥40 years were initially identified. After excluding 2626 individuals (1633 with incomplete ocular disease data, 583 with missing CDAI data, and 410 with incomplete covariate information), 4455 individuals remained for the final analysis (Fig. 1). To evaluate potential selection bias, baseline characteristics were compared between included and excluded participants (Supplementary Table S1).

### Assessment of Composite Dietary Antioxidant Index

Dietary intake was assessed using two nonconsecutive 24-hour recalls: the first at a mobile examination center and the second by telephone three to 10 days later. Six dietary antioxidants (vitamins A, C, and E, zinc, selenium, and carotenoids) were included, excluding supplements, medications, and drinking water. Overall dietary antioxidant exposure was quantified using a modified CDAI based on Wright's method.^15^ Each antioxidant intake was standardized (subtracting the mean and dividing by the SD), and the six standardized values were equally weighted and summed to derive the CDAI score.^16^

$$\begin{array}{c} CDAI=\sum_{n=1}^{6}\left(\frac{x_{n}-\mu_{n}}{s_{n}}\right) \end{array}$$

In this formula, *x_n_* represents the daily intake of antioxidant components; *µ_n_* denotes the mean of *x_n_*; and *s_n_* signifies the standard deviation for *µ_n__._*

### Assessment of FI

Frailty was evaluated using the FI, with >0.21 indicating frailty.^17^^,^^18^ The FI was constructed according to a widely adopted deficit accumulation framework, with items selected based on established criteria: deficits were required to reflect health status, their prevalence generally increased with age, they were not overly prevalent in the population, and they collectively covered multiple physiological systems.^19^ All deficits had >80% data completeness, consistent with prior NHANES-based frailty studies.^20^

The FI included 49 variables across six domains: physical function, social and economic function, mental health and cognition, chronic diseases, laboratory measures, and self-rated health and healthcare use (Supplementary Table S2). To avoid circularity, ocular disease outcomes were not included. Some FI components—such as diabetes and cardiovascular conditions—overlap conceptually with known ocular risk factors; these were retained to capture the participant's overall health deficit burden.

Each deficit was scored 0–1, and the FI was calculated as the proportion of present deficits. Subdomain scores were calculated similarly. This approach allows a comprehensive assessment of overall frailty, reflecting cumulative deficits rather than any single domain.

### Assessment of Major Ocular Diseases

In NHANES 2005–2008, participants aged ≥40 years underwent 45° non-mydriatic fundus photography using a Canon CR6-45NM camera (Canon Inc., Tokyo, Japan). Trained technicians followed standardized protocols, and images were graded independently by at least two certified evaluators from the University of Wisconsin. When both eyes were available, the eye with the more severe condition was analyzed.

DR was defined as the presence of ≥1 microaneurysm or dot hemorrhage on retinal images in participants with diabetes, based on the NHANES summary variable using a simplified ETDRS-based grading protocol. Retinopathy referred to any type of retinopathy in at least one eye. AMD included early (drusen and/or pigmentary abnormalities) and late (exudative changes or geographic atrophy) stages. Imaging outcomes (retinopathy, AMD, and DR participants with diabetes) were assessed via fundus photography. Cataract was identified by a self-reported history of cataract surgery, and glaucoma by a self-reported physician diagnosis.

Participants with AMD or retinopathy confirmed on fundus imaging were classified as “any objectively confirmed ocular disease,” whereas those with any of the four conditions (DR, AMD, cataract, or glaucoma) were defined as “any ocular disease.” These definitions have been broadly applied in previous research.^21^^,^^22^

### Covariates

Consistent with prior studies, we adjusted for key demographic and lifestyle covariates to reduce confounding. These included age (years), sex (male or female, based on biological sex at birth), ethnicity (Mexican American, non-Hispanic Black, non-Hispanic White, other Hispanic, or other/multi-racial), education (less than high school, high school, or above), marital status (married/living with partner, widowed/divorced/separated, never married), family income-to-poverty ratio (PIR: low <1.3, medium 1.3–3.5, high ≥3.5), smoking status (never, former, current), and alcohol consumption (yes/no).

### Statistical Analysis

All analyses accounted for the complex multistage design of NHANES using sample weights, strata (SDMVSTRA), and primary sampling units (SDMVPSU). Two-day dietary weights (wtdr2d) were applied, and multi-year weights for 2005–2008 were derived by dividing the two-year weight by the number of combined cycles. Continuous variables are presented as weighted means ± standard deviations (SD) and compared using one-way analysis of variance, whereas categorical variables are expressed as weighted percentages and compared using χ^2^ tests. Three models were constructed: a crude model, Model 1 adjusted for age and sex, and Model 2 further adjusted for all confounders. All main and subgroup analyses were conducted under the same survey weighting scheme.

Weighted multivariable logistic regression was used to examine associations of CDAI and FI with major ocular diseases, with restricted cubic splines (RCS) used to explore potential nonlinear relationships. Missing values in the six FI subdomains were addressed using multiple imputations by chained equations with 20 imputations and 50 iterations. The relative contribution of CDAI components and FI subdomains was quantified using the Lindeman–Merenda–Gold (LMG) metric. For each imputed dataset, survey-weighted logistic regression models were fitted, standardized coefficients were pooled across imputations using Rubin's rules, and contributions were calculated on the logit (linear predictor) scale. This analysis was performed for descriptive and comparative purposes; formal confidence intervals were not calculated, and results should be interpreted as exploratory.

To assess joint effects, CDAI was dichotomized at 0 (representing average dietary antioxidant intake) and FI at 0.21. Cross-classification produced four subgroups, with low FI + high CDAI as the reference, and fully adjusted logistic regression models (Model 3) were used to estimate combined associations with ocular outcomes.

Sensitivity analyses were conducted in two ways: first, using unweighted models and alternative multiple imputations by chained equations imputations for FI subdomains to assess the robustness of the findings; second, for DR analyses, the FI was recalculated after excluding diabetes status and HbAlc to evaluate whether these components influenced the observed associations. In addition, subgroup analyses by demographic and lifestyle factors were performed to explore possible effect heterogeneity.

All statistical analyses were performed using R software (version 4.2.2). All tests were two-sided, and a *P* value < 0.05 was considered statistically significant.

## Results

### Population Baseline Characteristics

Among the 4455 participants, 1326 (29.76%) had any ocular disease; 827 (18.56%) had any objectively confirmed ocular disease; 539 (12.10%) had any retinopathy; 333 (7.47%) had AMD; 558 (12.53%) had cataracts; 241 (5.41%) had DR; and 256 (5.75%) had glaucoma. Participants with any ocular disease tended to have higher age, lower educational attainment, lower poverty income ratio, higher smoking rates, higher FI scores, and lower CDAI scores than participants without ocular disease (Table 1). Baseline characteristics stratified by other specific ocular outcomes are presented in the Supplementary Materials (Supplementary Tables S3–S8).

**Table 1.** Baseline Characteristics of Participants

Characteristic	Overall (*n* = 4455)	No Ocular Disease (*n* = 3129)	Any Ocular Disease (*n* = 1326)	*P* Value
Sex				0.132
Male	2194 (45%)	1501 (45%)	693 (47%)	
Female	2261 (55%)	1628 (55%)	633 (53%)	
Age	56.54 (11.59)	53.99 (10.06)	64.67 (12.39)	**<0.001**
Ethnicity				0.347
Mexican American	634 (4.9%)	478 (5.0%)	156 (4.5%)	
Non-Hispanic Black	887 (9.2%)	618 (9.0%)	269 (10%)	
Non-Hispanic White	2524 (79%)	1726 (79%)	798 (80%)	
Other Hispanic	289 (2.7%)	216 (2.7%)	73 (2.8%)	
Other race—including multi-racial	121 (3.9%)	91 (4.2%)	30 (3.0%)	
Educational level				**<0.001**
Below high school	1212 (16%)	784 (14%)	428 (22%)	
High school	1111 (27%)	768 (26%)	343 (30%)	
Above high school	2132 (57%)	1577 (60%)	555 (48%)	
PIR				**<0.001**
Low	1075 (15%)	753 (15%)	322 (16%)	
Medium	1704 (34%)	1069 (30%)	635 (48%)	
High	1676 (51%)	1307 (56%)	369 (36%)	
Marital status				**<0.001**
Married or living with partner	2901 (69%)	2092 (70%)	809 (63%)	
Widowed	498 (8.3%)	237 (5.5%)	261 (17%)	
Divorced or separated	759 (17%)	568 (18%)	191 (13%)	
Never married	297 (6.2%)	232 (6.4%)	65 (5.7%)	
History of smoking				**<0.001**
Never	2095 (48%)	1506 (50%)	589 (44%)	
Former	1475 (31%)	943 (29%)	532 (37%)	
Current	885 (20%)	680 (21%)	205 (19%)	
Drinking				**<0.001**
Yes	3052 (73%)	2207 (75%)	845 (65%)	
No	1403 (27%)	922 (25%)	481 (35%)	
FI	0.14 (0.09)	0.14 (0.08)	0.18 (0.10)	**<0.001**
CDAI	0.93 (3.86)	1.13 (3.91)	0.31 (3.61)	**<0.001**

Imaging-graded outcomes (retinopathy, AMD, and DR in participants with diabetes) were assessed from fundus photography, while self-reported/procedure outcomes (cataract surgery, glaucoma) were obtained from questionnaires or medical records. Continuous variable was presented as mean ± SD, and categorical variables as n (unweighted) (%).

### Associations of FI and CDAI With Major Ocular Diseases

Table 2 shows weighted multivariable logistic regression results adjusting sequentially for age, sex, and all relevant covariates. Both FI and CDAI were associated with major ocular diseases, with associations varying in direction and strength by disease type.

**Table 2.** Associations of CDAI and FI With Major Ocular Diseases^a^

		Crude Model		Model 1		Model 2	
Ocular Diseases	Character	OR (95%CI)	*P* Value	OR (95%CI)	*P* Value	OR (95%CI)	P Value
Any ocular disease							
	CDAI	0.94 (0.92–0.97)	**<0.001**	0.96 (0.93–0.99)	**0.004**	0.97 (0.94–1.00)	**0.031**
	FI (Per 1-SD increase)	1.53 (1.41–1.67)	**<0.001**	1.41 (1.29–1.54)	**<0.001**	1.35 (1.23–1.47)	**<0.001**
Any objectively confirmed ocular disease							
	CDAI	0.96 (0.94–0.99)	**0.017**	0.98 (0.95–1.01)	0.117	0.99 (0.97–1.02)	0.599
	FI (Per 1-SD increase)	1.41 (1.27–1.57)	**<0.001**	1.32 (1.18–1.47)	**<0.001**	1.23 (1.09–1.39)	**0.002**
Any retinopathy							
	CDAI	0.96 (0.93–0.99)	**0.013**	0.97 (0.94–1.00)	**0.035**	0.99 (0.96–1.01)	0.320
	FI (Per 1-SD increase)	1.42 (1.26–1.60)	**<0.001**	1.41 (1.25–1.59)	**<0.001**	1.32 (1.16–1.50)	**<0.001**
AMD							
	CDAI	0.98 (0.93–1.02)	0.296	1.01 (0.96–1.06)	0.841	1.01 (0.96–1.07)	0.692
	FI (Per 1-SD increase)	1.35 (1.21–1.50)	**<0.001**	1.13 (1.01–1.26)	**0.033**	1.08 (0.96–1.22)	**0.178**
DR							
	CDAI	0.94 (0.89–1.00)	0.058	0.95 (0.89–1.02)	0.144	0.98 (0.92–1.04)	0.424
	FI (Per 1-SD increase)	2.24 (2.02–2.49)	**<0.001**	2.23 (2.03–2.45)	**<0.001**	2.21 (1.95–2.49)	**<0.001**
Cataract							
	CDAI	0.93 (0.90–0.97)	**<0.001**	0.96 (0.92–1.00)	0.051	0.96 (0.92–1.00)	0.059
	FI (Per 1-SD increase)	1.64 (1.48–1.81)	**<0.001**	1.39 (1.22–1.58)	**<0.001**	1.40 (1.22–1.60)	**<0.001**
Glaucoma							
	CDAI	0.90 (0.84, 0.96)	**0.002**	0.91 (0.85–0.98)	**0.011**	0.91 (0.84–0.98)	**0.014**
	FI (Per 1-SD increase)	1.43 (1.24–1.65)	**<0.001**	1.32 (1.12–1.56)	**0.002**	1.34 (1.12–1.61)	**0.003**

Imaging-graded outcomes (retinopathy, AMD, and DR in participants with diabetes) were assessed from fundus photography, whereas self-reported/procedure outcomes (cataract surgery, glaucoma) were obtained from questionnaires or medical records.

a Crude model, unadjusted analysis; Model 1, adjusted for age and sex; Model 2, further adjusted for ethnicity, marital status, education level, poverty income ratio, smoking and alcohol consumption.

The FI consistently showed positive associations across all models. In Model 2, higher FI was significantly associated with greater odds of all ocular diseases, including any ocular disease [OR (95% confidence interval {CI}) = 1.35 (1.23–1.47)], any objectively confirmed ocular disease [OR (95% CI) = 1.23 (1.09–1.39)], any retinopathy [OR (95% CI) = 1.32 (1.16–1.50)], diabetic retinopathy [OR (95% CI) = 2.21 (1.95–2.49)], cataract [OR (95% CI) = 1.40 (1.22–1.60)], and glaucoma [OR (95% CI) = 1.34 (1.12–1.61)], all *P* < 0.05. In contrast, the associations between CDAI and ocular diseases varied by disease type. In Model 2, higher CDAI was associated with lower odds of any ocular disease [OR (95% CI) = 0.97 (0.94–1.00)] and glaucoma [OR (95% CI) = 0.91 (0.84–0.98)] (both *P* < 0.05). For cataract, higher CDAI showed a tendency toward lower odds [OR (95% CI) = 0.96 (0.92–1.00)], although his association was not statistically significant. No significant associations were observed between CDAI and other ocular outcomes.

Based on Model 2, RCS analyses indicated that lower CDAI scores were associated with lower odds of any ocular disease and cataract, showing approximately linear relationships, while no significant associations were observed for objectively confirmed ocular disease, retinopathy, DR, glaucoma, or AMD. Higher FI scores were associated with higher odds of any ocular disease, objectively confirmed ocular disease, retinopathy, cataract, DR, and glaucoma, with a non-linear association observed for DR; whereas no significant association was observed for AMD (Supplementary Figs. S1, S2). Overall, FI remained consistently associated with multiple ocular diseases, whereas higher CDAI, reflecting greater dietary antioxidant intake, appeared to be linked with lower odds of certain outcomes, particularly any ocular disease, glaucoma, and cataract.

### Contribution Analysis of Different Antioxidant Components and Frailty Assessment Domains to Major Ocular Diseases

**Figure 2.**
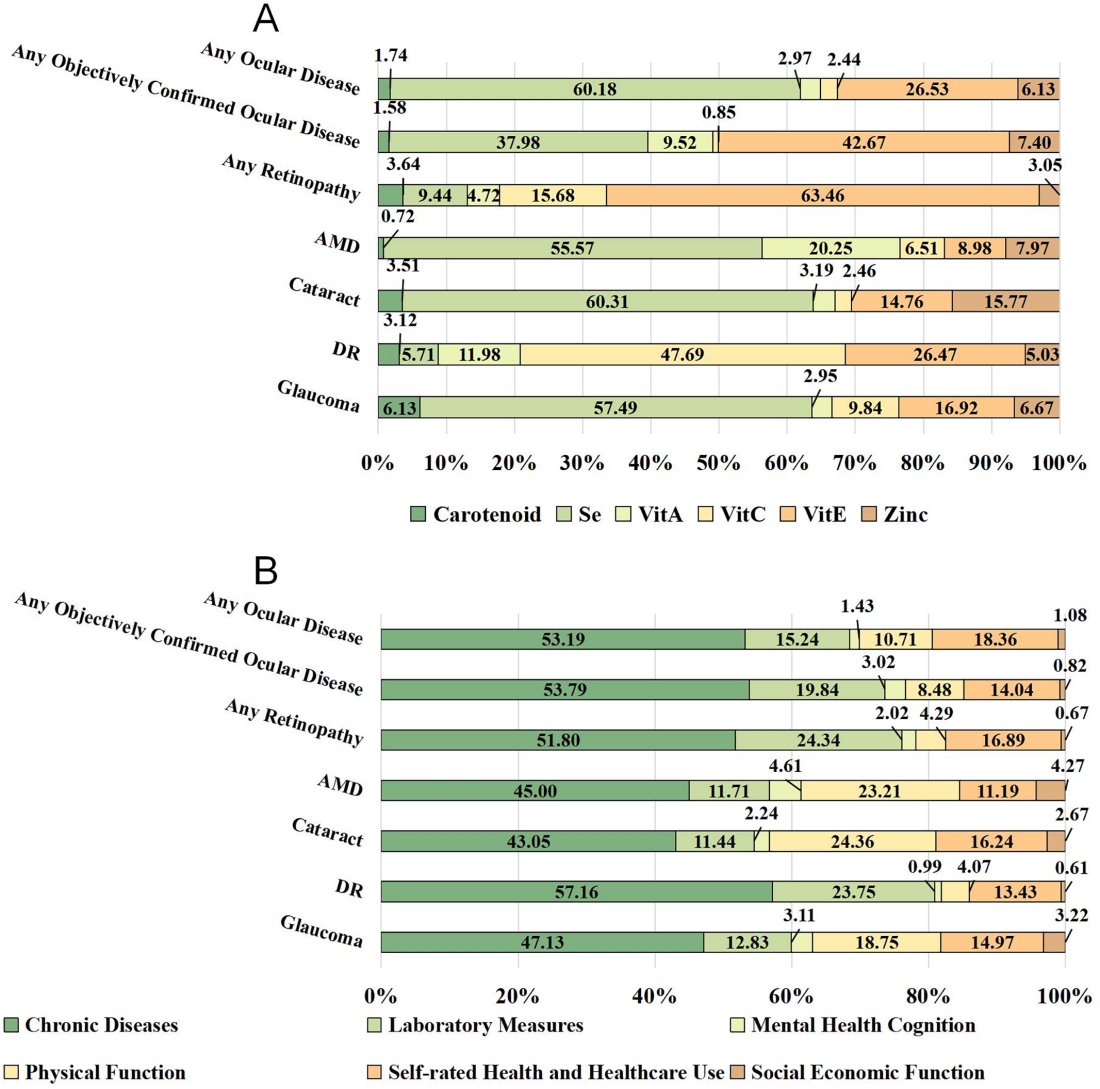
The relative contribution distribution of FI and CDAI domains to major ocular diseases. (**A**) Relative contribution of CDAI domains to major ocular diseases. (**B**) Relative contribution of FI domains to major ocular diseases. Imaging-graded outcomes (retinopathy, AMD, and DR in participants with diabetes) were assessed from fundus photography, whereas self-reported/procedure outcomes (cataract, glaucoma) were obtained from questionnaires or medical records. Component analyses are considered exploratory.

An exploratory LMG-based variable importance analysis assessed the relative contributions of CDAI components and FI domains to ocular diseases; given multiple comparisons, the results should be interpreted with caution. Contributions varied by disease. Among CDAI components, selenium (Se) and vitamin E (Vit E) showed the largest contributions—Se tended to contribute more to any ocular disease, AMD, cataract, and glaucoma, whereas Vit E tended to contribute more to any objectively confirmed ocular disease and retinopathy; vitamin C (Vit C) showed the largest contribution for DR. For FI domains, chronic disease burden tended to account for a larger share across outcomes, with other domains contributing variably by disease type (Fig. 2). Overall, CDAI and FI showed distinct patterns of relative contributions across ocular diseases, with selenium (Se), vitamin E (Vit E), and chronic disease burden (including heart failure, hypertension, diabetes, cancer, and kidney dysfunction) appearing among the more influential factors.

### Joint Effects of FI and CDAI on Major Ocular Diseases

**Figure 3.**
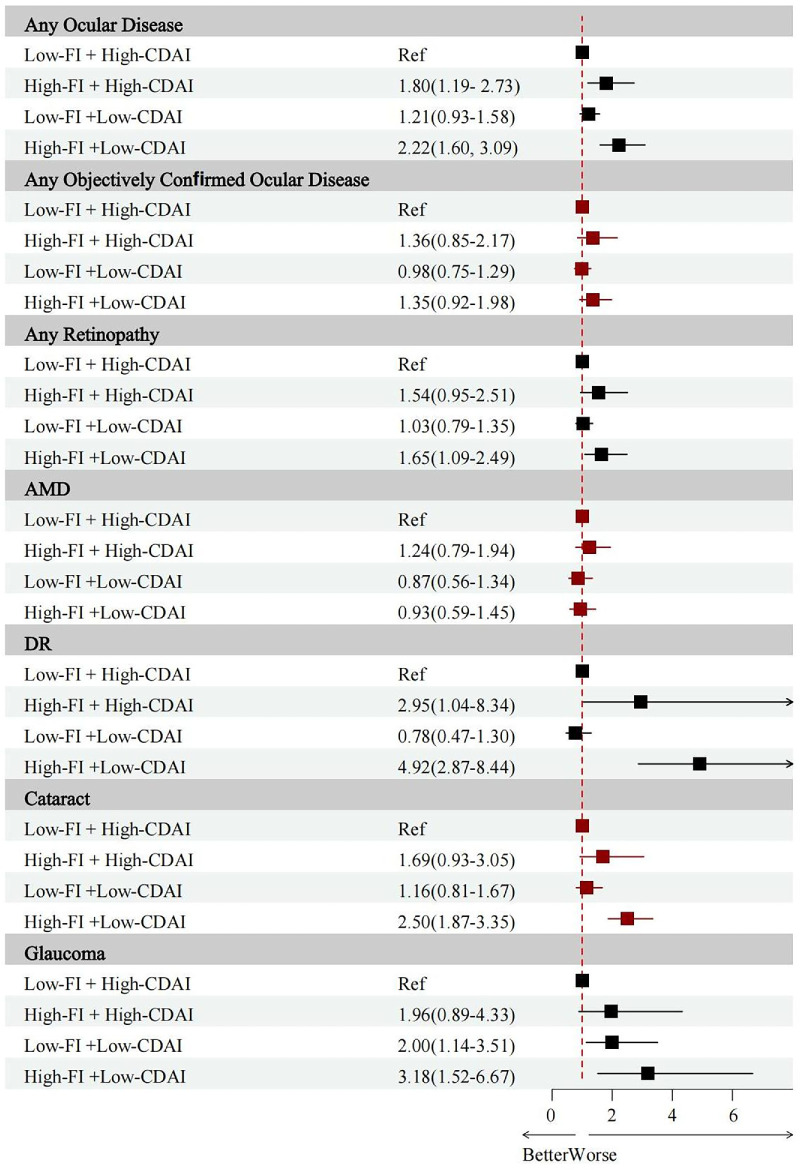
Joint effects of FI and CDAI on major ocular diseases. The results were based on Model 2, which is adjusted for age, sex, ethnicity, educational level, PIR, marital status, smoking, and drinking. Imaging-graded outcomes (retinopathy, AMD, and DR in participants with diabetes) were assessed from fundus photography, whereas self-reported/procedure outcomes (cataract, glaucoma) were obtained from questionnaires or medical records.

The joint associations of FI and CDAI were examined using paired combinations. Compared with the reference group (“low FI + high CDAI”), the “high FI + high CDAI” group showed higher odds for any ocular disease [OR (95% CI) = 1.80 (1.19–2.73)] and DR [OR (95% CI) = 2.95 (1.04–8.34)], with moderately elevated odds observed for any retinopathy [OR (95% CI) = 1.54 (0.95–2.51)] and cataract [OR (95% CI) = 1.69 (0.93–3.05)]. In contrast, the “high FI + low CDAI” group was associated with higher odds for most outcomes, including any ocular disease [OR (95% CI) = 2.22 (1.60–3.09)], any retinopathy [OR (95% CI) = 1.65 (1.09–2.49)], DR [OR (95% CI) = 4.92 (2.87–8.44)], cataract [OR (95% CI) = 2.50 (1.87–3.35)], and glaucoma [OR (95% CI) = 3.18 (1.52–6.67)]. The “low FI + low CDAI” group showed smaller increases in odds, most of which were not statistically significant, except for glaucoma [OR (95% CI) = 2.00 (1.14–3.51)] (Fig. 3).

Overall, frailty was consistently associated with higher odds of ocular diseases, whereas higher dietary antioxidant intake was linked to lower odds. The combination of frailty and low antioxidant intake was associated with the highest observed odds, with results depending on the dichotomized grouping and not reflecting a formal statistical interaction.

### Stratified and Sensitivity Analysis

Exploratory subgroup analyses were performed for each ocular outcome, considering age, sex, smoking status, alcohol consumption, PIR, and other covariates, and these results should be interpreted with caution in light of multiple comparisons. Potential interactions were noted between CDAI and alcohol consumption for any ocular disease, any objectively confirmed ocular disease, any retinopathy, and AMD (all *P* < 0.05), with odds slightly lower among alcohol consumers. Regarding frailty, FI interactions with sex for cataract (*P* < 0.05) suggested that women tended to show relatively larger associations at higher frailty levels, and interactions with PIR for DR (*P* < 0.05) suggested relatively stronger associations among higher-income participants (Supplementary Tables S9–S15). Overall, these results were consistent with the main analyses.

Sensitivity analyses using alternative multiple imputation methods for the FI subdomains showed that the contributions of individual FI domains to major ocular diseases remained largely consistent with the primary analysis (Supplementary Figure S3). Furthermore, recalculating FI for DR analyses after excluding diabetes and HbA1c items yielded results consistent with the primary analysis (Supplementary Table S16). These sensitivity analyses further confirmed the consistency of the observed associations.

## Discussion

This study examined the joint effects of the FI and CDAI on major ocular diseases and quantified the relative contributions of individual antioxidant components and frailty subdomains. FI and CDAI values showed opposite patterns across major ocular diseases. Based on dichotomized groups, participants with higher FI and lower CDAI values had the highest observed odds of major ocular diseases. Furthermore, the contributions of antioxidant components and frailty domains varied across different ocular outcomes. Se and Vit E consistently corresponded to lower observed odds across multiple eye diseases, whereas chronic disease burden showed the strongest associations among FI domains. Overall, these findings highlight the relevance of assessing frailty alongside dietary antioxidant status when describing ocular health patterns in older adults.

Oxidative stress plays a key role in the pathophysiology of the visual system.^23^ Dietary antioxidants may lower the risk of ocular diseases by reducing oxidative stress and inflammation.^5^ As a measure of overall dietary antioxidant potential, higher CDAI was associated with lower observed odds of “any ocular disease” and “glaucoma” in this study and showed a trend toward lower odds for “cataract,” consistent with previous reports.^24^^,^^25^ Notably, a cross-sectional study of 1706 individuals found that adherence to an antioxidant-rich diet tended to have lower odds of age-related macular degeneration [OR (95%CI) = 0.58 (0.33, 0.89)].^6^ However, we did not observe this association, possibly due to population differences or residual confounding. This study further showed that the contributions of individual antioxidant components to ocular diseases vary (exploratory component analyses; results should be interpreted with caution), potentially because of disease-specific oxidative stress mechanisms.^4^ Glaucoma, a neurodegenerative disorder characterized by retinal ganglion cells degeneration, is closely linked to oxidative stress.^26^ We found that participants with higher Se and Vit C intake tended to have the lowest odds of glaucoma. Specifically, Vit C reduces oxidative damage by scavenging free radicals, boosting neurotrophic factor expression, and improving mitochondrial function,^27^ whereas Se protects retinal ganglion cells by activating glutathione peroxidase and thioredoxin reductase systems.^28^ Cataracts primarily result from aggregation and impaired repair of lens proteins.^29^^,^^30^ We found that higher Zn and Se levels were linked to lower odds of cataracts. Se maintains lens homeostasis via selenoprotein-mediated antioxidant defense,^31^ whereas Zn, a cofactor of Cu/Zn-superoxide dismutase, stabilizes membranes and preserves redox balance.^32^^,^^33^ These findings suggest that considering the distinct roles of antioxidant components across ocular diseases may be valuable when designing targeted dietary strategies.

Besides dietary antioxidants, overall physiological reserve and functional decline are closely associated with ocular health. Our findings showed that higher FI scores were significantly associated with higher odds of multiple ocular diseases. For most outcomes, the associations were approximately linear; however, RCS analyses revealed a non-linear pattern for DR, indicating that individuals with moderate to severe frailty had higher observed odds. Evidence suggests a bidirectional association between frailty and ocular diseases. NHANES-based studies reported that DR [OR (95% CI) = 4.25 (1.08–16.67)], unilateral or bilateral visual field defects [OR (95% CI) = 2.07 (1.42–3.02) and 1.74 (1.20–2.52), respectively], and near vision impairment [OR (95% CI) = 4.5 (1.7–12.7)] were all associated with higher frailty scores. Genetic analyses indicated that DR was associated with higher frailty scores [β (95% CI) = 0.03 (0.01–0.05)], whereas visual impairment tended to be linked to higher frailty, with potential contributions of limitations in physical activity and social participation.^11^^,^^12^^,^^20^ Moreover, a recent Mendelian randomization study identified significant genetic correlations between FI and ten ocular traits, suggesting a potential association with conditions such as cataract, keratitis, and vitreous disorders.^34^ Contribution analysis indicated that the “Chronic disease” domain accounted for the largest share of observed ocular disease patterns (exploratory component analyses; results should be interpreted with caution). Consistent with previous findings, a cross-sectional study including 4146 participants reported that lower cardiovascular health (reflected by lower LE8 scores) was associated with higher odds of several ocular diseases, including DR [OR (95% CI) = 10.23 (3.11–33.61)] and glaucoma [OR (95% CI) = 2.76 (1.47–5.21)].^21^ Chronic conditions, including diabetes and hypertension, may damage the retinal vasculature and optic nerve via microvascular injury, oxidative stress, and inflammation.^35^^,^^36^ These observations highlight the potential relevance of chronic disease detection and management for visual health.

 Although some FI components, such as diabetes and cardiovascular conditions, are conceptually related to established ocular risk factors, the FI captures overall multisystem health vulnerability rather than individual diseases or physiologic frailty. The observed associations therefore likely reflect the cumulative impact of overall health deficits rather than the effect of any single condition. Although residual confounding cannot be entirely ruled out, this perspective helps contextualize the relationship between FI and ocular outcomes.

Joint exposure analysis further showed that participants with high frailty and low dietary antioxidant potential exhibited the highest observed prevalence of ocular diseases, which may be associated with shared pathways involving oxidative stress and chronic inflammation. A previous NHANES study found that higher CDAI was associated with lower frailty prevalence [OR (95% CI) = 0.81 (0.70–0.93)], partially mediated by oxidative stress biomarkers such as serum albumin and uric acid (all *P* < 0.05).^14^ Similarly, a study of 21,354 adults aged ≥40 years reported that each one-unit increase in CDAI was associated with a 3.7% lower prevalence of frailty, with vitamin A, C, E, selenium, and carotenoids showing nonlinear J-shaped associations with frailty.^13^ These findings are consistent with the hypothesis that higher dietary antioxidant intake is associated with lower frailty levels and align with the cutoff-dependent combined associations of CDAI and FI observed in the study, which may indicate underlying systemic oxidative stress and inflammation. An elevated FI reflects cumulative physiological deficits and a pro-inflammatory state,^37^ whereas a lower CDAI indicates insufficient dietary antioxidant defense. The coexistence of these conditions may worsen microvascular damage and mitochondrial dysfunction,^38^^,^^39^ leading to oxidative injury in the retina, lens, and optic nerve. Antioxidants such as vitamins C and E, selenium, and carotenoids help counteract oxidative stress and inflammation,^40^ whereas frailty is closely linked to redox imbalance and accelerated cellular senescence.^41^ In this study, participants with both high frailty and low antioxidant potential showed higher observed prevalence of ocular diseases, potentially reflecting systemic vulnerability.

Stratified analyses indicated that the associations of CDAI and FI with ocular diseases varied across populations (these are exploratory subgroup analyses with multiple comparisons and should be interpreted cautiously). The association of CDAI with ocular diseases appeared weaker among alcohol consumers, consistent with a cross-sectional study in China, possibly reflecting diminished dietary antioxidant effects associated with alcohol-induced oxidative stress.^42^ FI interacted significantly with sex for cataract, with stronger observed associations in women, potentially linked to estrogen changes and heightened systemic inflammation sensitivity.^43^^,^^44^ Additionally, FI interacted with PIR in DR, with the observed association between frailty and DR stronger in higher-income individuals. This contrasts with prior reports suggesting lower-income populations are more vulnerable due to higher chronic disease burden,^45^ possibly because DR diagnosis in this study relied on retinal imaging, and higher-income individuals are more likely to undergo fundus examinations, which could amplify the observed effect.^46^ These findings indicate that the associations between CDAI, FI, and ocular diseases vary across populations (these exploratory subgroup analyses warrant cautious interpretation), highlighting the potential value of tailored risk assessment and population-specific considerations.

Excluded participants were generally older, frailer, had lower socioeconomic status, and lower CDAI scores, which may reflect greater difficulty in completing dietary and ocular assessments. Most ocular disease outcomes were similarly distributed between included and excluded participants, and the remaining sample size was large, providing context for interpreting the study findings.

The novelty of this study lies in the comprehensive evaluation of the independent and joint effects of CDAI and FI on multiple major ocular diseases, together with variable importance analyses clarifying the relative contributions of individual dietary antioxidant components and FI domains. Several limitations should be acknowledged. First, the cross-sectional design precludes causal inference. Second, cataract and glaucoma were ascertained using self-reported surgery or physician diagnosis without structural or functional confirmation, which may introduce ascertainment bias and result in underestimation of disease prevalence and nondifferential misclassification, potentially biasing associations toward the null. In addition, some joint-exposure subgroups yielded large effect estimates with wide confidence intervals and should be interpreted as exploratory. Dietary assessment relied on 24-hour recalls, which may not capture long-term dietary patterns, and the exclusion of certain plant-derived bioactive antioxidants may have introduced measurement errors. Finally, as the data were derived from NHANES 2005–2008, changes in population characteristics, dietary patterns, and diagnostic practices may limit direct generalizability to contemporary populations. Nevertheless, frailty and dietary antioxidant status represent fundamental and persistent determinants of health, and the ocular diseases examined remain clinically relevant under current diagnostic standards. Therefore large-scale prospective and experimental studies incorporating clinical ophthalmic assessments are needed to further validate our findings.

## Conclusions

Our findings indicate that FI and CDAI are independently associated with higher and lower odds of major ocular diseases, respectively. Participants with both high frailty and low dietary antioxidant intake showed the highest observed odds of ocular disease. Variable importance analysis showed that contributions of antioxidant components and FI domains differed by ocular outcomes, based on dichotomized FI and CDAI groups. These findings highlight the potential value of frailty status and dietary antioxidant profiles for identifying individuals at higher risk of specific ocular diseases and for informing future preventive research. Future prospective cohort studies and mechanistic investigations are warranted to establish causality and elucidate underlying biological pathways.

## Supplementary Material

Supplement 1
