# Supplementary material for: Associations of Frailty Index and Composite Dietary Antioxidant Index With Major Ocular Diseases and Analysis of Component Contributions: A Cross-Sectional Study
Source: Transl Vis Sci Technol. 2026 Apr 27;15(4):25. doi: 10.1167/tvst.15.4.25 (PMC13112495; doi:10.1167/tvst.15.4.25)
Supplement: Supplement 1 [file tvst-15-4-25_s001.docx]

**Supplementary material**

**Table S1** Baseline characteristics of included and excluded participants

**Table S2.** The 49-item frailty index framework used in this study and related variables in the National Health and Nutrition Examination Survey.

**Table S3.** Baseline characteristics of the study population by any objectively confirmed ocular disease

**Table S4.** Baseline characteristics of the study population by any retinopathy

**Table S5.** Baseline characteristics of the study population by AMD

**Table S6.** Baseline characteristics of the study population by cataract

**Table S7.** Baseline characteristics of the study population by DR

**Table S8.** Baseline characteristics of the study population by glaucoma

**Table S9** Subgroup analysis of the relationships of CDAI and FI with the risk of any ocular disease

**Table S10** Subgroup analysis of the relationships of CDAI and FI with the risk of any objectively confirmed ocular disease

**Table S11** Subgroup analysis of the relationships of CDAI and FI with the risk of any retinopathy

**Table S12** Subgroup analysis of the relationships of CDAI and FI with the risk of AMD

**Table S13** Subgroup analysis of the relationships of CDAI and FI with the risk of cataract

**Table S14** Subgroup analysis of the relationships of CDAI and FI with the risk of DR

**Table S15** Subgroup analysis of the relationships of CDAI and FI with the risk of glaucoma

**Table S16** Associations of FI for sensitivity analysis with DR in weighted NHANES

**Figure. S1** Restricted cubic spline plots of the association between CDAI and major ocular diseases

**Figure. S2** Restricted cubic spline plots of the association between FI and major ocular diseases

**Figure. S3** Radar plots comparing the absolute contribution values between the main analysis and sensitivity analysis

| **Table S1.Baseline characteristics of included and excluded participants** | | | | |
| --- | --- | --- | --- | --- |
| **Variable** | **Total** | **Missing** | **Non-missing** | ***P* value** |
| Number (n) | 7081 | 2626 | 4455 |  |
| Sex |  |  |  |  |
| Male | 3506 (50%) | 1312 (50%) | 2194(49%) | 0.562 |
| Female | 3575 (50%) | 1314 (50%) | 2261(51%) |  |
| Age | 60.63 (12.99) | 62.77 (13.79) | 59.36(12.33) | **<0.001** |
| Ethnicity |  |  |  |  |
| Mexican American | 1103(16%) | 469 (18%) | 634(14%) | **<0.001** |
| Non-Hispanic Black | 1514(21%) | 627 (24%) | 887(20%) |  |
| Non-Hispanic White | 3688(52%) | 1164 (44%) | 2524(57%) |  |
| Other Hispanic | 518(7%) | 229 (9%) | 289(6%) |  |
| Other Race - Including Multi-Racial | 258(4%) | 137 (5%) | 121(3%) |  |
| Educational Level |  |  |  |  |
| Below highschool | 1141 (16%) | 599 (23%) | 1212(27%) | **<0.001** |
| Highschool | 2830 (40%) | 1049 (40%) | 1111(25%) |  |
| Above highschool | 3097 (44%) | 965 (37%) | 2132(48%) |  |
| PIR |  |  |  |  |
| Low | 1747(27%) | 672 (33%) | 1075(24%) | **<0.001** |
| Medium | 2555(39%) | 851 (41%) | 1704(38%) |  |
| High | 2208(34%) | 532 (26%) | 1676(38%) |  |
| Marriage |  |  |  |  |
| Married or Living with partner | 3449 (63%) | 548 (53%) | 2901(65%) | **<0.001** |
| Widowed | 737 (13%) | 239 (23%) | 498(11%) |  |
| Divorced or Separated | 945 (17%) | 186 (18%) | 759(17%) |  |
| Never Married | 366 (7%) | 69 (7%) | 297(7%) |  |
| Smoking |  |  |  |  |
| never | 3433 (49%) | 1338 (51%) | 2095(47%) | **0.002** |
| former | 2248 (32%) | 773 (30%) | 1475(33%) |  |
| current | 1392 (20%) | 507 (19%) | 885(20%) |  |
| Drinking |  |  |  |  |
| Yes | 4198 (67%) | 1146(64%) | 3052(69%) | **0.001** |
| No | 2046 (33%) | 643(36%) | 1403(31%) |  |
| FI | 0.17 (0.11) | 0.19 (0.013) | 0.16(0.10) | **<0.001** |
| CDAI | 0.31 (3.73) | -0.14 (3.54) | 0.44(3.78) | **<0.001** |
| Cataract |  |  |  |  |
| No | 4897 (88%) | 1000 (88%) | 3897 (87%) | 0.551 |
| Yes | 692 (12%) | 134 (12%) | 558 (13%) |  |
| Glaucoma |  |  |  |  |
| No | 5241 (94%) | 1042 (94%) | 4199 (94%) | 0.402 |
| Yes | 328 (6%) | 72 (6%) | 256 (6%) |  |
| DR |  |  |  |  |
| No | 5176 (95%) | 962 (95%) | 4214 (95%) | 1.000 |
| Yes | 296 (5%) | 55 (5%) | 241 (5%) |  |
| AMD |  |  |  |  |
| No | 5158 (92%) | 1036 (91%) | 4122 (93%) | 0.107 |
| Yes | 435 (8%) | 102 (9%) | 333 (7%) |  |
| Any Retinopathy |  |  |  |  |
| No | 4920 (88%) | 1004 (88%) | 3916 (88%) | 0.804 |
| Yes | 673 (12%) | 134 (12%) | 539 (12%) |  |
| Any Ocular Disease |  |  |  |  |
| No | 3814 (68%) | 685 (60%) | 3129 (70%) | **<0.001** |
| Yes | 1779 (32%) | 453 (40%) | 1326 (30%) |  |
| Any Objectively Confirmed Ocular Disease |  |  |  |  |
| No | 4542 (81%) | 914 (80%) | 3628 (81%) | 0.412 |
| Yes | 1051 (19%) | 224 (20%) | 827 (19%) |  |

Note: AMD, age-related macular degeneration; PIR, poverty income ratio; DR, diabetic retinopathy. FI, frailty index; CDAI, composite dietary antioxidant index. Imaging-graded outcomes (retinopathy, AMD, and DR in participants with diabetes) were assessed from fundus photography, while self-reported/procedure outcomes (cataract, glaucoma) were obtained from questionnaires or medical records.

Sample sizes vary across variables due to missing data. Statistics are based on available data for each variable.

Continuous variables are reported as means ± SD , and categorical variables as n (unweighted) (%).

| **Table S2. The 49-item frailty index framework used in this study and related variables in the National Health and Nutrition Examination Survey** | | |
| --- | --- | --- |
| Item | Variables in NHANES | Score |
| **Social Economic Function** | | |
| 1. Difficulty managing money | pfq060a,pfq061a | no difficulty=0; |
|  |  | some difficulty=0.33; |
|  |  | much difficulty=0.66; |
|  |  | unable to do=1 |
| 2. Difficulty attending social events | pfq060r,pfq061r | unable to do=1 |
| 3. Difficulty performing leisure activity at home | pfq060s,pfq061s | unable to do=1 |
| **Physical Function** | | |
| 4. Difficulty walking for a quarter mile | pfq060b,pfq061b | unable to do=1 |
| 5. Difficulty walking up 10 steps | pfq060c,pfq061c | unable to do=1 |
| 6. Difficulty stooping, crouching, kneeling | pfq060d,pfq061d | unable to do=1 |
| 7. Difficulty lifting or carrying | pfq060e,pfq061e | unable to do=1 |
| 8. Difficulty performing house chores | pfq060f,pfq061f | unable to do=1 |
| 9. Difficulty preparing meals | pfq060g,pfq061g | unable to do=1 |
| 10. Difficulty standing up from armless chair | pfq060i,pfq061i | unable to do=1 |
| 11. Difficulty getting in and out of bed | pfq060j,pfq061j | unable to do=1 |
| 12. Difficulty using fork, knife, drinking from a cup | pfq060k,pfq061k | unable to do=1 |
| 13. Difficulty dressing yourself | pfq060l,pfq061l | unable to do=1 |
| 14. Difficulty standing for long periods | pfq060m,pfq061m | unable to do=1 |
| 15. Difficulty grasping/holding small objects | pfq060p,pfq061p | unable to do=1 |
| 16. Difficulty pushing or pulling large objects | pfq061t | unable to do=1 |
| **Mental Health Cognition** | | |
| 17. Having little interest in doing things | ciqd008,ciqd009,dpq010 | nearly every day = 1 |
|  |  | more than half the days = 0.66 |
|  |  | several days = 0.33 |
| 18. Feeling down, depressed, or hopeless | dpq020,ciqd001,ciqd002 | the same to above |
| 19. Having trouble sleeping or sleeping too much | dpq030,ciqd025,ciqd026 | nearly every day = 1 |
|  |  | more than half the days = 0.66 |
|  |  | several days = 0.33 |
| 20. Feeling tired or having little energy | dpq040 | nearly every day = 1 |
|  |  | more than half the days = 0.66 |
|  |  | several days = 0.33 |
| 21. Poor appetite or overeating | ciqd019,ciqd022,dpq050 | nearly every day = 1 |
|  |  | more than half the days = 0.66 |
|  |  | several days = 0.33 |
| 22. Feeling bad about yourself | dpq060,ciqd029 | nearly every day = 1 |
|  |  | more than half the days = 0.66 |
|  |  | several days = 0.33 |
| 23. Having trouble concentrating on things | dpq070,ciqd043 | nearly every day = 1 |
|  |  | more than half the days = 0.66 |
|  |  | several days = 0.33 |
| 24. Experience confusion/memory problems | pfq056,pfq057 | yes=1; no=0 |
| **Chronic Diseases** | | |
| 25. Has a doctor ever told you that you have arthritis? | mcq160a | yes = 1; no = 0 |
| 26. Has a doctor ever told you that you have thyroid problems? | mcq160i,mcd160m,mcq160m | yes = 1; no = 0 |
| 27. Has a doctor ever told you that you have chronic bronchitis? | mcq160k,mcq160p | yes = 1; no = 0 |
| 28. Has a doctor ever told you that you have cancer or malignancy? | mcq220 | yes = 1; no = 0 |
| 29. Has a doctor ever told you that you have congestive heart failure? | mcq160b | yes = 1; no = 0 |
| 30. Has a doctor ever told you that you have coronary heart disease? | mcq160c | yes = 1; no = 0 |
| 31. Has a doctor ever told you that you have angina/angina pectoris? | mcq160d | yes = 1; no = 0 |
| 32. Has a doctor ever told you that you had heart attack? | mcq160e | yes = 1; no = 0 |
| 33. Has a doctor ever told you that you had a stroke? | mcq160f | yes = 1; no = 0 |
| 34. Has a doctor ever told you that you have high blood pressure? | bpq020 | yes = 1; no = 0 |
| 35. Has a doctor ever told you that you have diabetes? | diq010 | yes = 1; no =0; borderline=0.5 |
| 36. Has a doctor ever told you that you have weak/failing kidneys? | kiq020,kiq022 | yes = 1; no =0 |
| 37. Does urine leakage bother you? | kiq040,kiq050 | greatly = 1 |
|  |  | very much = 0.75 |
|  |  | somewhat = 0.5 |
|  |  | only a little = 0.25 |
| **Self-rated Health & Healthcare Use** | | |
| 38. General health condition | huq010 | excellent, very good, good = 0 fair, poor = 1 |
| 39. Your health now compared with 1 year ago | huq020 | about the same, better = 0 worse = 1 |
| 40. Overnight hospital patient in the last year | huq070,hud070,huq071 | yes = 1, no = 0 |
| 41. Number of times receive healthcare over past year | huq050,huq051 | none = 0; 1-4 = 0.5; >=5 = 1 |
| 42. Number of prescription medicines taken | rxd030,rxduse,rxd295,rxdcount | no = 0; 1-4 = 0.5; >=5 = 1 |
| **Laboratory Measures** | | |
| 43. Body mass index (kg/m^2) | bmxbmi | <18.5, ≥30 = 1 25–<30 = 0.5 18.5–25 = 0 |
| 44. Glycosylated hemoglobin (%) | lbxgh | 0%–5.7% = 0, >5.7% = 1 |
| 45. Red blood cell count (million cells/uL) | lbxrbcsi | M: 4.7–6. 1 = 0, Other = 1 F: 4.2–5.4 = 0, Other = 1 |
| 46. Hemoglobin (g/dL) | lbxhgb | M: 13.5–18 = 0, Other = 1 F: 12–16 = 0, Other = 1 |
| 47. Red cell distribution width (%) | lbxrdw | 11.6–14.6 = 0, Other = 1 |
| 48. Lymphocyte percent (%) | lbxlypct | 20–40 = 0, Other = 1 |
| 49. Segmented neutrophil percentage (%) | lbxnepct | 40–80 = 0, Other = 1 |
| NHANES, National Health and Nutrition Examination Survey. | | |
| *These seven indicators of depression symptoms were based on the Patient Health Questionnaire-9. | | |

| **Table S3. Baseline characteristics of the study population by any objectively confirmed ocular disease** | | | |
| --- | --- | --- | --- |
| **Characteristic** | **Any Objectively Confirmed Ocular Disease** | | |
|  | **NO (n = 3628)** | **Yes (n = 827)** | **P value** |
| Sex |  |  |  |
| Male | 1,750 (44%) | 444 (50%) | **0.008** |
| Female | 1,878 (56%) | 383 (50%) |  |
| Age | 55.41 (10.97) | 63.00 (12.86) | **<0.001** |
| Ethnicity |  |  |  |
| Mexican American | 519 (4.7%) | 115 (5.6%) | **0.023** |
| Non-Hispanic Black | 707 (9.0%) | 180 (11%) |  |
| Non-Hispanic White | 2,057 (79%) | 467 (78%) |  |
| Other Hispanic | 240 (2.6%) | 49 (3.5%) |  |
| Other Race - Including Multi-Racial | 105 (4.2%) | 16 (1.9%) |  |
| Educational Level |  |  |  |
| Below highschool | 937 (15%) | 275 (23%) | **<0.001** |
| Highschool | 891 (26%) | 220 (30%) |  |
| Above highschool | 1,800 (59%) | 332 (47%) |  |
| Poverty Income Ratio |  |  |  |
| Low | 860 (14%) | 215 (18%) | **<0.001** |
| Medium | 1,314 (32%) | 390 (48%) |  |
| High | 1,454 (54%) | 222 (35%) |  |
| Marriage |  |  |  |
| Married or Living with partner | 2,400 (70%) | 501 (63%) | **<0.001** |
| Widowed | 346 (7.0%) | 152 (16%) |  |
| Divorced or Separated | 631 (17%) | 128 (15%) |  |
| Never Married | 251 (6.2%) | 46 (6.3%) |  |
| Smoking |  |  |  |
| never | 1,714 (49%) | 381 (44%) | 0.185 |
| former | 1,177 (31%) | 298 (33%) |  |
| current | 737 (20%) | 148 (23%) |  |
| Drinking |  |  |  |
| Yes | 2,527 (74%) | 525 (66%) | **<0.001** |
| No | 1,101 (26%) | 302 (34%) |  |
| FI | 0.14 (0.09) | 0.17 (0.10) | **<0.001** |
| CDAI | 1.01 (3.87) | 0.50 (3.76) | **0.012** |

Note: PIR, poverty income ratio; FI, frailty index; CDAI, composite dietary antioxidant index. Continuous variables are reported as means ± SD, and categorical variables as n (unweighted) (%).

| **Table S4. Baseline characteristics of the study population by any rsetinopathy** | | | |
| --- | --- | --- | --- |
| **Characteristic** | **Any Retinopathy** | | |
|  | **NO (n = 3916)** | **Yes (n = 539)** | **P value** |
| Sex |  |  |  |
| Male | 1,894 (44%) | 300 (55%) | **<0.001** |
| Female | 2,022 (56%) | 239 (45%) |  |
| Age | 56.19 (11.47) | 59.95 (12.25) | **<0.001** |
| Ethnicity |  |  |  |
| Mexican American | 549 (4.6%) | 85 (7.0%) | **<0.001** |
| Non-Hispanic Black | 730 (8.6%) | 157 (15%) |  |
| Non-Hispanic White | 2,274 (80%) | 250 (71%) |  |
| Other Hispanic | 253 (2.5%) | 36 (4.6%) |  |
| Other Race - Including Multi-Racial | 110 (4.1%) | 11 (1.9%) |  |
| Educational Level |  |  |  |
| Below highschool | 1,017 (15%) | 195 (25%) | **<0.001** |
| Highschool | 974 (26%) | 137 (31%) |  |
| Above highschool | 1,925 (58%) | 207 (44%) |  |
| Poverty Income Ratio |  |  |  |
| Low | 931 (15%) | 144 (18%) | **<0.001** |
| Medium | 1,456 (33%) | 248 (45%) |  |
| High | 1,529 (53%) | 147 (37%) |  |
| Marriage |  |  |  |
| Married or Living with partner | 2,560 (69%) | 341 (66%) | 0.136 |
| Widowed | 422 (8.1%) | 76 (11%) |  |
| Divorced or Separated | 676 (17%) | 83 (14%) |  |
| Never Married | 258 (5.9%) | 39 (9.2%) |  |
| Smoking |  |  |  |
| never | 1,838 (48%) | 257 (47%) | **0.015** |
| former | 1,301 (32%) | 174 (26%) |  |
| current | 777 (20%) | 108 (27%) |  |
| Drinking |  |  |  |
| Yes | 2,715 (74%) | 337 (65%) | **<0.001** |
| No | 1,201 (26%) | 202 (35%) |  |
| FI | 0.14 (0.09) | 0.18 (0.11) | **<0.001** |
| CDAI | 0.98 (3.86) | 0.45 (3.83) | **0.003** |

Note: PIR, poverty income ratio; FI, frailty index; CDAI, composite dietary antioxidant index. Continuous variables are reported as means ± SD, and categorical variables as n (unweighted) (%).

| **Table S5. Baseline characteristics of the study population by AMD** | | | |
| --- | --- | --- | --- |
| **Characteristic** | **AMD** | | |
|  | **NO (n = 4122)** | **Yes (n = 333 )** | **P value** |
| Sex |  |  |  |
| Male | 2,029 (45%) | 165 (42%) | **<0.001** |
| Female | 2,093 (55%) | 168 (58%) |  |
| Age | 55.73 (11.11) | 68.56 12.02) | **<0.001** |
| Ethnicity |  |  |  |
| Mexican American | 597 (4.9%) | 37 (3.5%) | **<0.001** |
| Non-Hispanic Black | 859 (9.6%) | 28 (3.5%) |  |
| Non-Hispanic White | 2,275 (79%) | 249 (90%) |  |
| Other Hispanic | 275 (2.8%) | 14 (1.7%) |  |
| Other Race - Including Multi-Racial | 116 (4.0%) | 5 (1.8%) |  |
| Educational Level |  |  |  |
| Below highschool | 1,114 (16%) | 98 (21%) | 0.187 |
| Highschool | 1,018 (27%) | 93 (28%) |  |
| Above highschool | 1,990 (57%) | 142 (51%) |  |
| Poverty Income Ratio |  |  |  |
| Low | 995 (15%) | 80 (16%) | **<0.001** |
| Medium | 1,536 (33%) | 168 (54%) |  |
| High | 1,591 (53%) | 85 (30%) |  |
| Marriage |  |  |  |
| Married or Living with partner | 2,719 (70%) | 182 (58%) | **<0.001** |
| Widowed | 408 (7.2%) | 90 (25%) |  |
| Divorced or Separated | 706 (17%) | 53 (16%) |  |
| Never Married | 289 (6.5%) | 8 (1.4%) |  |
| Smoking |  |  |  |
| never | 1,950 (49%) | 145 (40%) | **0.003** |
| former | 1,335 (31%) | 140 (43%) |  |
| current | 837 (21%) | 48 (17%) |  |
| Drinking |  |  |  |
| Yes | 2,834 (73%) | 218 (67%) | 0.082 |
| No | 1,288 (27%) | 115 (33%) |  |
| FI | 0.14 (0.09) | 0.17 (0.10) | **<0.001** |
| CDAI | 0.96 (3.88) | 0.60 (3.58) | 0.334 |

Note: AMD, age-related macular degeneration; PIR, poverty income ratio; FI, frailty index; CDAI, composite dietary antioxidant index. Continuous variables are reported as means ± SD, and categorical variables as n (unweighted) (%).

| **Table S6. Baseline characteristics of the study population by cataract** | | | |
| --- | --- | --- | --- |
| **Characteristic** | **Cataract** | | |
|  | **NO (n = 3897)** | **Yes (n = 558)** | **P value** |
| Sex |  |  |  |
| Male | 1,939 (46%) | 255 (37%) | **<0.001** |
| Female | 1,958 (54%) | 303 (63%) |  |
| Age | 54.91 (10.50) | 72.39 (9.63) | **<0.001** |
| Ethnicity |  |  |  |
| Mexican American | 596 (5.1%) | 38 (2.5%) | **0.006** |
| Non-Hispanic Black | 819 (9.7%) | 68 (5.0%) |  |
| Non-Hispanic White | 2,112 (78%) | 412 (87%) |  |
| Other Hispanic | 260 (2.8%) | 29 (1.8%) |  |
| Other Race - Including Multi-Racial | 110 (3.9%) | 11 (3.6%) |  |
| Educational Level |  |  |  |
| Below highschool | 1,026 (15%) | 186 (25%) | **<0.001** |
| Highschool | 962 (26%) | 149 (29%) |  |
| Above highschool | 1,909 (58%) | 223 (45%) |  |
| Poverty Income Ratio |  |  |  |
| Low | 941 (15%) | 134 (17%) | **<0.001** |
| Medium | 1,412 (32%) | 292 (54%) |  |
| High | 1,544 (53%) | 132 (29%) |  |
| Marriage |  |  |  |
| Married or Living with partner | 2,579 (70%) | 322 (58%) | **<0.001** |
| Widowed | 342 (6.3%) | 156 (28%) |  |
| Divorced or Separated | 693 (17%) | 66 (11%) |  |
| Never Married | 283 (6.5%) | 14 (3.2%) |  |
| Smoking |  |  |  |
| never | 1,845 (48%) | 250 (47%) | **<0.001** |
| former | 1,221 (30%) | 254 (43%) |  |
| current | 831 (22%) | 54 (9.8%) |  |
| Drinking |  |  |  |
| Yes | 2,717 (74%) | 335 (61%) | **<0.001** |
| No | 1,180 (26%) | 223 (39%) |  |
| FI | 0.14 (0.09) | 0.19 (0.11) | **<0.001** |
| CDAI | 1.02 (3.89) | 0.10 (3.40) | **<0.001** |

Note: PIR, poverty income ratio; FI, frailty index; CDAI, composite dietary antioxidant index. Continuous variables are reported as means ± SD, and categorical variables as n (unweighted) (%).

| **Table S7. Baseline characteristics of the study population by DR** | | | |
| --- | --- | --- | --- |
| **Characteristic** | **DR** | | |
|  | **NO (n = 4214)** | **Yes (n = 241)** | **P value** |
| Sex |  |  |  |
| Male | 2,055 (45%) | 139 (55%) | **0.019** |
| Female | 2,159 (55%) | 102 (45%) |  |
| Age | 56.31 (11.54) | 63.01 (11.34) | **<0.001** |
| Ethnicity |  |  |  |
| Mexican American | 595 (4.8%) | 39 (6.7%) | **<0.001** |
| Non-Hispanic Black | 808 (8.9%) | 79 (20%) |  |
| Non-Hispanic White | 2,421 (80%) | 103 (66%) |  |
| Other Hispanic | 273 (2.6%) | 16 (6.6%) |  |
| Other Race - Including Multi-Racial | 117 (4.0%) | 4 (1.0%) |  |
| Educational Level |  |  |  |
| Below highschool | 1,115 (16%) | 97 (31%) | **<0.001** |
| Highschool | 1,055 (27%) | 56 (25%) |  |
| Above highschool | 2,044 (57%) | 88 (44%) |  |
| Poverty Income Ratio |  |  |  |
| Low | 1,000 (15%) | 75 (24%) | **<0.001** |
| Medium | 1,597 (34%) | 107 (44%) |  |
| High | 1,617 (52%) | 59 (31%) |  |
| Marriage |  |  |  |
| Married or Living with partner | 2,749 (69%) | 152 (67%) | **0.011** |
| Widowed | 461 (8.1%) | 37 (14%) |  |
| Divorced or Separated | 720 (17%) | 39 (16%) |  |
| Never Married | 284 (6.3%) | 13 (3.4%) |  |
| Smoking |  |  |  |
| never | 1,979 (48%) | 116 (49%) | 0.766 |
| former | 1,392 (31%) | 83 (33%) |  |
| current | 843 (21%) | 42 (18%) |  |
| Drinking |  |  |  |
| Yes | 2,913 (73%) | 139 (55%) | **<0.001** |
| No | 1,301 (27%) | 102 (45%) |  |
| FI | 0.14 (0.09) | 0.24 (0.11) | **<0.001** |
| CDAI | 0.96 (3.86) | 0.20 (3.62) | **0.012** |

Note: PIR, poverty income ratio; DR, diabetic retinopathy; FI, frailty index; CDAI, composite dietary antioxidant index. Continuous variables are reported as means ± SD, and categorical variables as n (unweighted) (%).

| **Table S8. Baseline characteristics of the study population by glaucoma** | | | |
| --- | --- | --- | --- |
| **Characteristic** | **Glaucoma** | | |
|  | **NO (n = 4199)** | **Yes (n = 256)** | **P value** |
| Sex |  |  |  |
| Male | 2,062 (45%) | 132 (49%) | 0.437 |
| Female | 2,137 (55%) | 124 (51%) |  |
| Age | 56.16 (11.43) | 64.24 (12.13) | **<0.001** |
| Ethnicity |  |  |  |
| Mexican American | 608 (4.9%) | 26 (3.4%) | 0.231 |
| Non-Hispanic Black | 822 (9.0%) | 65 (14%) |  |
| Non-Hispanic White | 2,381 (79%) | 143 (76%) |  |
| Other Hispanic | 273 (2.8%) | 16 (2.0%) |  |
| Other Race - Including Multi-Racial | 115 (3.8%) | 6 (4.6%) |  |
| Educational Level |  |  |  |
| Below highschool | 1,135 (16%) | 77 (19%) | 0.135 |
| Highschool | 1,043 (26%) | 68 (32%) |  |
| Above highschool | 2,021 (57%) | 111 (48%) |  |
| Poverty Income Ratio |  |  |  |
| Low | 1,016 (15%) | 59 (14%) | 0.053 |
| Medium | 1,585 (34%) | 119 (44%) |  |
| High | 1,598 (52%) | 78 (43%) |  |
| Marriage |  |  |  |
| Married or Living with partner | 2,744 (69%) | 157 (64%) | **0.009** |
| Widowed | 445 (7.8%) | 53 (18%) |  |
| Divorced or Separated | 724 (17%) | 35 (12%) |  |
| Never Married | 286 (6.2%) | 11 (6.3%) |  |
| Smoking |  |  |  |
| never | 1,981 (48%) | 114 (42%) | **0.016** |
| former | 1,364 (31%) | 111 (44%) |  |
| current | 854 (21%) | 31 (14%) |  |
| Drinking |  |  |  |
| Yes | 2,883 (73%) | 169 (69%) | 0.306 |
| No | 1,316 (27%) | 87 (31%) |  |
| FI | 0.14 (0.09) | 0.18 (0.11) | **<0.001** |
| CDAI | 1.00 (3.88) | -0.30 (3.07) | **0.002** |

Note: PIR, poverty income ratio; FI, frailty index; CDAI, composite dietary antioxidant index. Continuous variables are reported as means ± SD, and categorical variables as n (unweighted) (%).

| **Table S9 Subgroup analysis of the relationships of CDAI and FI with the risk of any ocular disease** | | | | |
| --- | --- | --- | --- | --- |
| **Subgroups** | **CDAI** | | **FI (Per 1-SD increase)** | |
|  | **OR (95%CI)** | **P Value** | **OR (95%CI)** | **P Value** |
| **Age** |  |  |  |  |
| **<65** | 0.97 (0.94, 0.99) | **0.011** | 1.34 (1.21, 1.48) | **<0.001** |
| **≥65** | 0.96 (0.93, 0.99) | **0.024** | 1.51 (1.34, 1.70) | **<0.001** |
| **P for trend** | 0.429 |  | 0.129 |  |
| **Sex** |  |  |  |  |
| **Male** | 0.96 (0.93, 0.99) | **0.003** | 1.39 (1.25, 1.55) | **<0.001** |
| **Female** | 0.99 (0.96, 1.02) | 0.480 | 1.45 (1.31, 1.62) | **<0.001** |
| **P for trend** | 0.376 |  | 0.101 |  |
| **Ethnicity** |  |  |  |  |
| Mexican American | 0.93 (0.87, 1.00) | **0.041** | 1.29 (1.05, 1.60) | **0.016** |
| Non-Hispanic Black | 0.99 (0.95, 1.04) | 0.817 | 1.50 (1.29, 1.75) | **<0.001** |
| Non-Hispanic White | 0.98 (0.96, 1.01) | 0.191 | 1.38 (1.25, 1.53) | **<0.001** |
| Other Hispanic | 0.91 (0.82, 1.01) | 0.064 | 1.52 (1.17, 1.99) | **0.002** |
| Other Race - Including Multi-Racial | 0.90 (0.75, 1.08) | 0.270 | 1.30 (0.78, 2.16) | 0.314 |
| **P for trend** | 0.145 |  | 0.737 |  |
| **Educational Level** |  |  |  |  |
| Below highschool | 0.95 (0.91, 0.99) | **0.020** | 1.38 (1.22, 1.56) | **<0.001** |
| Highschool | 0.99 (0.96, 1.03) | 0.700 | 1.39 (1.20, 1.60) | **<0.001** |
| Above highschool | 0.97 (0.94, 1.01) | 0.110 | 1.44 (1.28, 1.63) | **<0.001** |
| **P for trend** | 0.359 |  | 0.754 |  |
| **PIR** |  |  |  |  |
| Low | 0.97 (0.93, 1.01) | 0.200 | 1.33 (1.17, 1.50) | **<0.001** |
| Medium | 0.97 (0.94, 1.00) | **0.050** | 1.46 (1.30, 1.63) | **<0.001** |
| High | 0.99 (0.95, 1.02) | 0.440 | 1.39 (1.18, 1.63) | **<0.001** |
| **P for trend** | 0.910 |  | 0.730 |  |
| **Marriage** |  |  |  |  |
| Married or Living with partner | 0.98 (0.95, 1.00) | 0.082 | 1.43 (1.30, 1.58) | **<0.001** |
| Widowed | 0.99 (0.93, 1.04) | 0.605 | 1.53 (1.25, 1.88) | **<0.001** |
| Divorced or Separated | 0.96 (0.91, 1.01) | 0.130 | 1.32 (1.11, 1.57) | **0.002** |
| Never Married | 0.96 (0.88, 1.04) | 0.341 | 1.32 (0.99, 1.74) | 0.057 |
| **P for trend** | 0.763 |  | 0.844 |  |
| **Smoking** |  |  |  |  |
| never | 1.00 (0.97, 1.03) | 0.977 | 1.45 (1.29, 1.63) | **<0.001** |
| former | 0.96 (0.93, 1.00) | **0.046** | 1.36 (1.20, 1.55) | **<0.001** |
| current | 0.94 (0.90, 0.99) | 0.014 | 1.41 (1.21, 1.63) | **<0.001** |
| **P for trend** | 0.097 |  | 0.763 |  |
| **Drinking** |  |  |  |  |
| No | 0.96 (0.93, 0.98) | **0.002** | 1.37 (1.24, 1.50) | **<0.001** |
| Yes | 1.00 (0.97, 1.04) | 0.793 | 1.48 (1.31, 1.67) | **<0.001** |
| **P for trend** | **0.029** |  | 0.399 |  |

Note: PIR, poverty income ratio; FI, frailty index; CDAI, composite dietary antioxidant index. The results were based on Model 2, which is adjusted for age, sex, ethnicity, educational level, PIR, marital status, smoking, and drinking. Subgroup analyses are considered exploratory.

| **Table S10. Subgroup analysis of the relationships of CDAI and FI with the risk of any objectively confirmed ocular disease** | | | | |
| --- | --- | --- | --- | --- |
| **Subgroups** | **CDAI** | | **FI (Per 1-SD increase)** | |
|  | **OR (95%CI)** | **P Value** | **OR (95%CI)** | **P Value** |
| **Age** |  |  |  |  |
| **<65** | 0.97 (0.95, 1.00) | 0.087 | 1.26 (1.14, 1.41) | **<0.001** |
| **≥65** | 1.00 (0.97, 1.04) | 0.933 | 1.31 (1.17, 1.47) | **<0.001** |
| **P for trend** | **0.041** |  | 0.779 |  |
| **Sex** |  |  |  |  |
| **Male** | 0.97 (0.94, 1.00) | 0.090 | 1.30 (1.16, 1.45) | **<0.001** |
| **Female** | 1.01 (0.98, 1.04) | 0.462 | 1.30 (1.16, 1.45) | **<0.001** |
| **P for trend** | 0.346 |  | 0.266 |  |
| **Ethnicity** |  |  |  |  |
| Mexican American | 0.93 (0.86, 1.00) | **0.046** | 1.30 (1.04, 1.62) | **0.022** |
| Non-Hispanic Black | 1.00 (0.95, 1.05) | 0.983 | 1.35 (1.15, 1.59) | **<0.001** |
| Non-Hispanic White | 1.01 (0.98, 1.04) | 0.465 | 1.23 (1.10, 1.37) | **<0.001** |
| Other Hispanic | 0.95 (0.85, 1.06) | 0.334 | 1.54 (1.14, 2.07) | **0.004** |
| Other Race - Including Multi-Racial | 0.95 (0.78, 1.15) | 0.593 | 1.16 (0.66, 2.04) | 0.615 |
| **P for trend** | 0.203 |  | 0.858 |  |
| **Educational Level** |  |  |  |  |
| Below highschool | 0.99 (0.95, 1.03) | 0.540 | 1.34 (1.18, 1.52) | **<0.001** |
| Highschool | 1.02 (0.98, 1.06) | 0.280 | 1.19 (1.02, 1.38) | **0.024** |
| Above highschool | 0.98 (0.94, 1.01) | 0.200 | 1.32 (1.16, 1.50) | **<0.001** |
| **P for trend** | 0.137 |  | 0.328 |  |
| **PIR** |  |  |  |  |
| Low | 0.99 (0.94, 1.03) | 0.570 | 1.24 (1.08, 1.41) | **0.002** |
| Medium | 1.00 (0.96, 1.03) | 0.900 | 1.30 (1.16, 1.46) | **<0.001** |
| High | 0.99 (0.95, 1.04) | 0.800 | 1.29 (1.08, 1.55) | **0.005** |
| **P for trend** | 0.870 |  | 0.889 |  |
| **Marriage** |  |  |  |  |
| Married or Living with partner | 1.00 (0.97, 1.02) | 0.765 | 1.30 (1.18, 1.44) | **<0.001** |
| Widowed | 1.00 (0.95, 1.06) | 0.906 | 1.47 (1.20, 1.79) | **<0.001** |
| Divorced or Separated | 0.97 (0.91, 1.03) | 0.255 | 1.20 (1.00, 1.44) | 0.055 |
| Never Married | 1.01 (0.93, 1.10) | 0.846 | 1.10 (0.80, 1.52) | 0.562 |
| **P for trend** | 0.619 |  | 0.564 |  |
| **Smoking** |  |  |  |  |
| never | 1.01 (0.98, 1.04) | 0.571 | 1.36 (1.20, 1.53) | **<0.001** |
| former | 1.00 (0.96, 1.04) | 0.923 | 1.20 (1.05, 1.38) | **0.007** |
| current | 0.96 (0.91, 1.01) | 0.086 | 1.32 (1.12, 1.54) | **0.001** |
| **P for trend** | 0.269 |  | 0.430 |  |
| **Drinking** |  |  |  |  |
| No | 0.97 (0.94, 0.99) | **0.018** | 1.24 (1.12, 1.37) | **<0.001** |
| Yes | 1.04 (1.00, 1.08) | **0.030** | 1.38 (1.22, 1.56) | **<0.001** |
| **P for trend** | **<0.001** |  | 0.435 |  |

Note: PIR, poverty income ratio; FI, frailty index; CDAI, composite dietary antioxidant index. The results were based on Model 2, which is adjusted for age, sex, ethnicity, educational level, PIR, marital status, smoking, and drinking. Subgroup analyses are considered exploratory.

| **Table S11. Subgroup analysis of the relationships of CDAI and FI with the risk of any retinopathy** | | | | |
| --- | --- | --- | --- | --- |
| **Subgroups** | **CDAI** | | **FI (Per 1-SD increase)** | |
|  | **OR (95%CI)** | **P Value** | **OR (95%CI)** | **P Value** |
| **Age** |  |  |  |  |
| **<65** | 0.97 (0.94, 1.00) | 0.075 | 1.28 (1.14, 1.44) | **<0.001** |
| **≥65** | 1.01 (0.96, 1.05) | 0.774 | 1.53 (1.33, 1.75) | **<0.001** |
| **P for trend** | 0.111 |  | 0.119 |  |
| **Sex** |  |  |  |  |
| **Male** | 0.98 (0.94, 1.01) | 0.190 | 1.34 (1.19, 1.51) | **<0.001** |
| **Female** | 1.00 (0.96, 1.04) | 0.854 | 1.42 (1.25, 1.61) | **<0.001** |
| **P for trend** | 0.985 |  | 0.155 |  |
| **Ethnicity** |  |  |  |  |
| Mexican American | 0.95 (0.88, 1.03) | 0.225 | 1.42 (1.11, 1.81) | **0.005** |
| Non-Hispanic Black | 0.99 (0.94, 1.04) | 0.579 | 1.39 (1.18, 1.65) | **<0.001** |
| Non-Hispanic White | 1.01 (0.98, 1.05) | 0.494 | 1.34 (1.18, 1.53) | **<0.001** |
| Other Hispanic | 0.89 (0.78, 1.01) | 0.078 | 1.56 (1.12, 2.17) | **0.009** |
| Other Race - Including Multi-Racial | 1.04 (0.83, 1.29) | 0.737 | 1.05 (0.53, 2.08) | 0.9 |
| **P for trend** | 0.297 |  | 0.976 |  |
| **Educational Level** |  |  |  |  |
| Below highschool | 0.98 (0.93, 1.03) | 0.360 | 1.46 (1.27, 1.68) | **<0.001** |
| Highschool | 1.03 (0.99, 1.08) | 0.150 | 1.25 (1.05, 1.49) | **0.014** |
| Above highschool | 0.96 (0.92, 1.01) | 0.090 | 1.41 (1.21, 1.64) | **<0.001** |
| **P for trend** | 0.052 |  | 0.319 |  |
| **PIR** |  |  |  |  |
| Low | 0.98 (0.93, 1.03) | 0.410 | 1.26 (1.08, 1.46) | **0.003** |
| Medium | 0.99 (0.95, 1.03) | 0.550 | 1.43 (1.26, 1.63) | **<0.001** |
| High | 1.00 (0.95, 1.05) | 0.890 | 1.44 (1.17, 1.76) | **<0.001** |
| **P for trend** | 0.950 |  | 0.356 |  |
| **Marriage** |  |  |  |  |
| Married or Living with partner | 0.99 (0.96, 1.02) | 0.484 | 1.38 (1.23, 1.54) | **<0.001** |
| Widowed | 1.00 (0.93, 1.08) | 0.945 | 1.74 (1.36, 2.21) | **<0.001** |
| Divorced or Separated | 0.98 (0.92, 1.05) | 0.641 | 1.26 (1.02, 1.56) | **0.035** |
| Never Married | 0.99 (0.90, 1.08) | 0.788 | 1.13 (0.80, 1.59) | 0.477 |
| **P for trend** | 0.944 |  | 0.383 |  |
| **Smoking** |  |  |  |  |
| never | 0.97 (0.94, 1.01) | 0.178 | 1.48 (1.29, 1.69) | **<0.001** |
| former | 1.02 (0.97, 1.07) | 0.443 | 1.33 (1.13, 1.55) | **<0.001** |
| current | 0.98 (0.93, 1.04) | 0.545 | 1.28 (1.07, 1.54) | **0.007** |
| **P for trend** | 0.445 |  | 0.343 |  |
| **Drinking** |  |  |  |  |
| No | 0.97 (0.93, 1.00) | 0.047 | 1.32 (1.18, 1.48) | **<0.001** |
| Yes | 1.02 (0.98, 1.07) | 0.305 | 1.48 (1.29, 1.71) | **<0.001** |
| **P for trend** | **0.011** |  | 0.358 |  |

Note: PIR, poverty income ratio; FI, frailty index; CDAI, composite dietary antioxidant index. The results were based on Model 2, which is adjusted for age, sex, ethnicity, educational level, PIR, marital status, smoking, and drinking. Subgroup analyses are considered exploratory.

| **Table S12. Subgroup analysis of the relationships of CDAI and FI with the risk of AMD** | | | | |
| --- | --- | --- | --- | --- |
| **Subgroups** | **CDAI** | | **FI (Per 1-SD increase)** | |
|  | **OR (95%CI)** | **P Value** | **OR (95%CI)** | **P Value** |
| **Age** |  |  |  |  |
| **<65** | 1.01 (0.95, 1.06) | 0.811 | 1.22 (0.98, 1.50) | 0.069 |
| **≥65** | 1.00 (0.96, 1.04) | 0.920 | 1.08 (0.93, 1.24) | 0.313 |
| **P for trend** | 0.916 |  | 0.408 |  |
| **Sex** |  |  |  |  |
| **Male** | 0.98 (0.93, 1.03) | 0.360 | 1.19 (1.01, 1.40) | **0.041** |
| **Female** | 1.04 (0.99, 1.09) | 0.099 | 1.08 (0.92, 1.28) | 0.348 |
| **P for trend** | 0.150 |  | 0.859 |  |
| **Ethnicity** |  |  |  |  |
| Mexican American | 0.89 (0.78, 1.01) | 0.076 | 1.14 (0.79, 1.63) | 0.492 |
| Non-Hispanic Black | 1.08 (0.99, 1.18) | 0.079 | 1.11 (0.76, 1.61) | 0.601 |
| Non-Hispanic White | 1.01 (0.97, 1.05) | 0.610 | 1.10 (0.95, 1.26) | 0.196 |
| Other Hispanic | 1.15 (0.95, 1.40) | 0.156 | 1.35 (0.80, 2.27) | 0.256 |
| Other Race - Including Multi-Racial | 0.75 (0.48, 1.17) | 0.206 | 1.14 (0.37, 3.47) | 0.820 |
| **P for trend** | 0.074 |  | 0.692 |  |
| **Educational Level** |  |  |  |  |
| Below highschool | 1.01 (0.94, 1.08) | 0.800 | 1.06 (0.86, 1.30) | 0.601 |
| Highschool | 1.00 (0.95, 1.07) | 0.880 | 1.11 (0.89, 1.38) | 0.367 |
| Above highschool | 1.01 (0.96, 1.06) | 0.710 | 1.18 (0.97, 1.43) | 0.091 |
| **P for trend** | 0.992 |  | 0.691 |  |
| **PIR** |  |  |  |  |
| Low | 1.01 (0.94, 1.09) | 0.760 | 1.23 (1.00, 1.50) | **0.047** |
| Medium | 1.02 (0.97, 1.07) | 0.520 | 0.99 (0.84, 1.18) | 0.949 |
| High | 1.01 (0.94, 1.08) | 0.870 | 1.19 (0.90, 1.56) | 0.217 |
| **P for trend** | 0.820 |  | 0.100 |  |
| **Marriage** |  |  |  |  |
| Married or Living with partner | 1.01 (0.97, 1.06) | 0.557 | 1.09 (0.92, 1.28) | 0.326 |
| Widowed | 1.02 (0.95, 1.10) | 0.508 | 1.31 (1.02, 1.68) | **0.032** |
| Divorced or Separated | 0.97 (0.88, 1.07) | 0.537 | 1.02 (0.77, 1.36) | 0.878 |
| Never Married | 1.18 (0.92, 1.49) | 0.189 | 0.87 (0.33, 2.26) | 0.769 |
| **P for trend** | 0.579 |  | 0.680 |  |
| **Smoking** |  |  |  |  |
| never | 1.08 (1.03, 1.14) | **0.001** | 1.08 (0.90, 1.30) | 0.415 |
| former | 0.95 (0.89, 1.01) | 0.108 | 1.05 (0.86, 1.28) | 0.617 |
| current | 0.94 (0.85, 1.03) | 0.187 | 1.30 (1.01, 1.68) | 0.038 |
| **P for trend** | **0.004** |  | 0.194 |  |
| **Drinking** |  |  |  |  |
| No | 0.98 (0.94, 1.02) | 0.358 | 1.14 (0.98, 1.33) | 0.080 |
| Yes | 1.07 (1.01, 1.14) | **0.022** | 1.09 (0.90, 1.32) | 0.367 |
| **P for trend** | **0.012** |  | 0.613 |  |

Note: AMD, age-related macular degeneration; PIR, poverty income ratio; FI, frailty index; CDAI, composite dietary antioxidant index. The results were based on Model 2, which is adjusted for age, sex, ethnicity, educational level, PIR, marital status, smoking, and drinking. Subgroup analyses are considered exploratory.

| **Table S13. Subgroup analysis of the relationships of CDAI and FI with the risk of cataract** | | | | |
| --- | --- | --- | --- | --- |
| **Subgroups** | **CDAI** | | **FI (Per 1-SD increase)** | |
|  | **OR (95%CI)** | **P Value** | **OR (95%CI)** | **P Value** |
| **Age** |  |  |  |  |
| **<65** | 0.95 (0.90, 1.02) | 0.146 | 1.36 (1.11, 1.66) | **0.003** |
| **≥65** | 0.94 (0.91, 0.98) | **0.001** | 1.46 (1.30, 1.65) | **<0.001** |
| **P for trend** | 0.977 |  | 0.539 |  |
| **Sex** |  |  |  |  |
| **Male** | 0.93 (0.88, 0.98) | **0.006** | 1.32 (1.14, 1.53) | **<0.001** |
| **Female** | 0.97 (0.93, 1.01) | 0.165 | 1.60 (1.38, 1.85) | **<0.001** |
| **P for trend** | 0.191 |  | **0.050** |  |
| **Ethnicity** |  |  |  |  |
| Mexican American | 1.01 (0.89, 1.13) | 0.925 | 1.44 (1.03, 2.03) | **0.035** |
| Non-Hispanic Black | 0.99 (0.90, 1.09) | 0.889 | 1.54 (1.18, 2.01) | **0.002** |
| Non-Hispanic White | 0.95 (0.92, 0.99) | **0.017** | 1.41 (1.24, 1.60) | **<0.001** |
| Other Hispanic | 0.84 (0.69, 1.02) | **0.072** | 1.67 (1.13, 2.47) | **0.010** |
| Other Race - Including Multi-Racial | 0.75 (0.48, 1.18) | 0.210 | 2.42 (0.76, 7.73) | 0.134 |
| **P for trend** | 0.244 |  | 0.947 |  |
| **Educational Level** |  |  |  |  |
| Below highschool | 0.96 (0.90, 1.02) | 0.160 | 1.36 (1.16, 1.60) | **<0.001** |
| Highschool | 0.95 (0.89, 1.01) | 0.100 | 1.57 (1.28, 1.94) | **<0.001** |
| Above highschool | 0.95 (0.90, 1.00) | 0.070 | 1.44 (1.21, 1.72) | **<0.001** |
| **P for trend** | 0.963 |  | 0.606 |  |
| **PIR** |  |  |  |  |
| Low | 0.97 (0.90, 1.04) | 0.410 | 1.47 (1.22, 1.78) | **<0.001** |
| Medium | 0.92 (0.88, 0.97) | **<0.001** | 1.41 (1.22, 1.63) | **<0.001** |
| High | 0.99 (0.93, 1.05) | 0.740 | 1.42 (1.11, 1.81) | **0.006** |
| **P for trend** | 0.180 |  | 0.770 |  |
| **Marriage** |  |  |  |  |
| Married or Living with partner | 0.94 (0.90, 0.98) | **0.005** | 1.43 (1.25, 1.64) | <0.001 |
| Widowed | 0.99 (0.93, 1.05) | 0.705 | 1.41 (1.13, 1.75) | **0.002** |
| Divorced or Separated | 0.99 (0.90, 1.08) | 0.750 | 1.44 (1.09, 1.90) | **0.010** |
| Never Married | 0.94 (0.77, 1.16) | 0.591 | 1.70 (0.93, 3.09) | 0.083 |
| **P for trend** | 0.601 |  | 0.843 |  |
| **Smoking** |  |  |  |  |
| never | 0.98 (0.94, 1.03) | 0.451 | 1.50 (1.28, 1.75) | **<0.001** |
| former | 0.93 (0.88, 0.98) | **0.007** | 1.33 (1.13, 1.57) | **0.001** |
| current | 0.92 (0.83, 1.02) | 0.104 | 1.67 (1.28, 2.17) | **<0.001** |
| **P for trend** | 0.165 |  | 0.382 |  |
| **Drinking** |  |  |  |  |
| No | 0.95 (0.91, 0.99) | **0.016** | 1.34 (1.17, 1.53) | **<0.001** |
| Yes | 0.96 (0.91, 1.02) | 0.172 | 1.60 (1.35, 1.88) | **<0.001** |
| **P for trend** | 0.930 |  | 0.071 |  |

Note: PIR, poverty income ratio; FI, frailty index; CDAI, composite dietary antioxidant index. The results were based on Model 2, which is adjusted for age, sex, ethnicity, educational level, PIR, marital status, smoking, and drinking. Subgroup analyses are considered exploratory.

| **Table S14. Subgroup analysis of the relationships of CDAI and FI with the risk of DR** | | | | |
| --- | --- | --- | --- | --- |
| **Subgroups** | **CDAI** | | **FI (Per 1-SD increase)** | |
|  | **OR (95%CI)** | **P Value** | **OR (95%CI)** | **P Value** |
| **Age** |  |  |  |  |
| **<65** | 0.97 (0.92, 1.02) | 0.194 | 2.01 (1.71, 2.36) | **<0.001** |
| **≥65** | 0.97 (0.91, 1.04) | 0.366 | 2.17 (1.82, 2.59) | **<0.001** |
| **P for trend** | 0.672 |  | 0.882 |  |
| **Sex** |  |  |  |  |
| **Male** | 0.96 (0.91, 1.01) | 0.142 | 2.15 (1.83, 2.53) | **<0.001** |
| **Female** | 0.98 (0.93, 1.05) | 0.619 | 2.00 (1.68, 2.39) | **<0.001** |
| **P for trend** | 0.942 |  | 0.814 |  |
| **Ethnicity** |  |  |  |  |
| Mexican American | 0.98 (0.87, 1.09) | 0.681 | 2.36 (1.69, 3.29) | **<0.001** |
| Non-Hispanic Black | 0.98 (0.91, 1.05) | 0.537 | 2.14 (1.72, 2.67) | **<0.001** |
| Non-Hispanic White | 0.99 (0.93, 1.05) | 0.693 | 2.05 (1.72, 2.44) | **<0.001** |
| Other Hispanic | 0.90 (0.75, 1.10) | 0.301 | 2.49 (1.56, 3.97) | **<0.001** |
| Other Race - Including Multi-Racial | 0.75 (0.28, 1.98) | 0.556 | 0.89 (0.12, 6.89) | 0.912 |
| **P for trend** | 0.857 |  | 0.926 |  |
| **Educational Level** |  |  |  |  |
| Below highschool | 0.93 (0.86, 1.00) | 0.060 | 1.99 (1.65, 2.39) | **<0.001** |
| Highschool | 1.01 (0.94, 1.09) | 0.800 | 1.96 (1.55, 2.49) | **<0.001** |
| Above highschool | 0.98 (0.92, 1.05) | 0.600 | 2.37 (1.93, 2.91) | **<0.001** |
| **P for trend** | 0.483 |  | 0.332 |  |
| **PIR** |  |  |  |  |
| Low | 0.91 (0.84, 0.99) | **0.030** | 1.75 (1.44, 2.14) | **<0.001** |
| Medium | 0.97 (0.91, 1.03) | 0.330 | 2.02 (1.70, 2.40) | **<0.001** |
| High | 1.03 (0.96, 1.10) | 0.460 | 3.22 (2.42, 4.30) | **<0.001** |
| **P for trend** | 0.090 |  | **0.040** |  |
| **Marriage** |  |  |  |  |
| Married or Living with partner | 0.97 (0.92, 1.02) | 0.216 | 2.15 (1.85, 2.50) | **<0.001** |
| Widowed | 1.02 (0.93, 1.13) | 0.624 | 2.56 (1.82, 3.61) | **<0.001** |
| Divorced or Separated | 0.96 (0.87, 1.07) | 0.472 | 1.87 (1.39, 2.52) | **<0.001** |
| Never Married | 0.95 (0.80, 1.14) | 0.587 | 2.05 (1.18, 3.54) | **0.011** |
| **P for trend** | 0.528 |  | 0.955 |  |
| **Smoking** |  |  |  |  |
| never | 0.96 (0.90, 1.02) | 0.207 | 2.28 (1.90, 2.74) | **<0.001** |
| former | 1.01 (0.94, 1.08) | 0.838 | 2.01 (1.64, 2.47) | **<0.001** |
| current | 0.95 (0.86, 1.04) | 0.255 | 1.92 (1.49, 2.48) | **<0.001** |
| **P for trend** | 0.429 |  | 0.475 |  |
| **Drinking** |  |  |  |  |
| No | 0.95 (0.90, 1.01) | 0.079 | 2.21 (1.89, 2.59) | **<0.001** |
| Yes | 1.00 (0.94, 1.06) | 0.899 | 1.95 (1.63, 2.33) | **<0.001** |
| **P for trend** | 0.178 |  | 0.229 |  |

Note: DR, diabetic retinopathy; PIR, poverty income ratio; FI, frailty index; CDAI, composite dietary antioxidant index. The results were based on Model 2, which is adjusted for age, sex, ethnicity, educational level, PIR, marital status, smoking, and drinking. Subgroup analyses are considered exploratory.

| **Table S15. Subgroup analysis of the relationships of CDAI and FI with the risk of glaucoma** | | | | |
| --- | --- | --- | --- | --- |
| **Subgroups** | **CDAI** | | **FI (Per 1-SD increase)** | |
|  | **OR (95%CI)** | **P Value** | **OR (95%CI)** | **P Value** |
| **Age** |  |  |  |  |
| **<65** | 0.92 (0.86, 0.98) | **0.015** | 1.59 (1.32, 1.92) | **<0.001** |
| **≥65** | 0.99 (0.94, 1.04) | 0.666 | 1.35 (1.16, 1.58) | **<0.001** |
| **P for trend** | 0.057 |  | 0.129 |  |
| **Sex** |  |  |  |  |
| **Male** | 0.94 (0.88, 1.00) | **0.038** | 1.42 (1.20, 1.69) | **<0.001** |
| **Female** | 0.98 (0.93, 1.04) | 0.484 | 1.44 (1.22, 1.71) | **<0.001** |
| **P for trend** | 0.437 |  | 0.581 |  |
| **Ethnicity** |  |  |  |  |
| Mexican American | 0.95 (0.82, 1.09) | 0.450 | 1.42 (0.96, 2.11) | 0.083 |
| Non-Hispanic Black | 0.96 (0.89, 1.05) | 0.383 | 1.57 (1.25, 1.99) | **<0.001** |
| Non-Hispanic White | 0.97 (0.92, 1.02) | 0.210 | 1.39 (1.18, 1.64) | **<0.001** |
| Other Hispanic | 1.01 (0.83, 1.23) | 0.897 | 1.61 (1.01, 2.57) | **0.044** |
| Other Race - Including Multi-Racial | 0.89 (0.60, 1.33) | 0.568 | 0.71 (0.21, 2.46) | 0.593 |
| **P for trend** | 0.898 |  | 0.799 |  |
| **Educational Level** |  |  |  |  |
| Below highschool | 0.95 (0.87, 1.03) | 0.180 | 1.46 (1.20, 1.78) | **<0.001** |
| Highschool | 0.98 (0.91, 1.06) | 0.580 | 1.45 (1.15, 1.84) | **0.002** |
| Above highschool | 0.96 (0.90, 1.02) | 0.220 | 1.38 (1.13, 1.68) | **0.001** |
| **P for trend** | 0.833 |  | 0.874 |  |
| **PIR** |  |  |  |  |
| Low | 0.95 (0.87, 1.03) | 0.230 | 1.74 (1.39, 2.16) | **<0.001** |
| Medium | 1.00 (0.95, 1.06) | 0.900 | 1.32 (1.10, 1.57) | **0.002** |
| High | 0.92 (0.85, 0.99) | 0.030 | 1.29 (0.99, 1.69) | 0.058 |
| **P for trend** | 0.150 |  | 0.076 |  |
| **Marriage** |  |  |  |  |
| Married or Living with partner | 0.99 (0.94, 1.04) | 0.609 | 1.36 (1.17, 1.60) | **<0.001** |
| Widowed | 0.95 (0.86, 1.04) | 0.287 | 1.33 (1.02, 1.74) | **0.034** |
| Divorced or Separated | 0.94 (0.84, 1.06) | 0.311 | 2.07 (1.49, 2.87) | **<0.001** |
| Never Married | 0.73 (0.54, 0.99) | **0.044** | 1.60 (0.82, 3.13) | 0.167 |
| **P for trend** | 0.153 |  | 0.093 |  |
| **Smoking** |  |  |  |  |
| never | 1.02 (0.96, 1.08) | 0.536 | 1.41 (1.18, 1.70) | **<0.001** |
| former | 0.91 (0.85, 0.97) | **0.007** | 1.38 (1.14, 1.68) | **0.001** |
| current | 0.93 (0.82, 1.04) | 0.207 | 1.54 (1.16, 2.05) | **0.003** |
| **P for trend** | 0.163 |  | 0.696 |  |
| **Drinking** |  |  |  |  |
| No | 0.96 (0.91, 1.01) | 0.094 | 1.53 (1.32, 1.78) | **<0.001** |
| Yes | 0.97 (0.90, 1.04) | 0.414 | 1.30 (1.07, 1.59) | **0.010** |
| **P for trend** | 0.855 |  | 0.308 |  |

Note: PIR, poverty income ratio; FI, frailty index; CDAI, composite dietary antioxidant index. The results were based on Model 2, which is adjusted for age, sex, ethnicity, educational level, PIR, marital status, smoking, and drinking. Subgroup analyses are considered exploratory.

| **Table S16. Associations of FI for sensitivity analysis with DR in weighted NHANES** | | | | | | | | |
| --- | --- | --- | --- | --- | --- | --- | --- | --- |
| Character | Crude Model | | | Model 1 | | | Model 2 | |
|  | OR (95%CI) | P Value | OR (95%CI) | | P Value | OR (95%CI) | | P Value |
| FI (Per 1-SD increase) | 1.78(1.60-1.99) | **< 0.001** | 1.76(1.60-1.93) | | **< 0.001** | 1.69(1.51-1.88) | | **< 0.001** |

Note: DR, diabetic retinopathy. FI was recalculated for DR analyses after removing diabetes and HbA1c items. Crude model: unadjusted; Model 1: adjusted for age and sex; Model 2: fully adjusted for age, sex, ethnicity, marital status, educational level, family income ratio, smoking, and drinking. OR, odds ratio; CI, confidence interval.


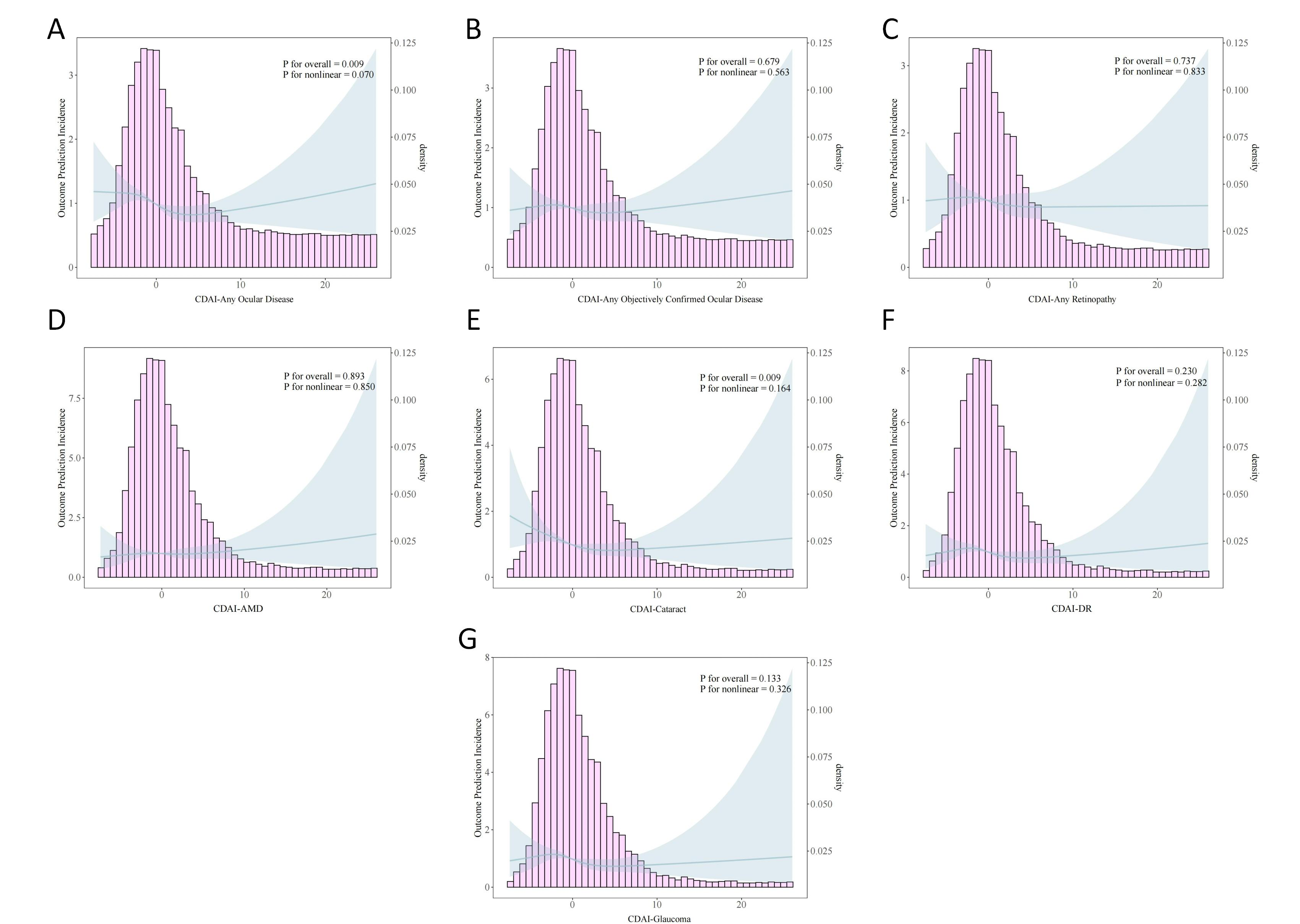


**Figure. S1** Restricted cubic spline plots of the association between CDAI and major ocular diseases

The results were based on Model 2, which is adjusted for age, sex, ethnicity, educational level, PIR, marital status, smoking, and drinking. Abbreviations: CDAI, composite dietary antioxidant index; AMD, age-related macular degeneration; DR, diabetic retinopathy.


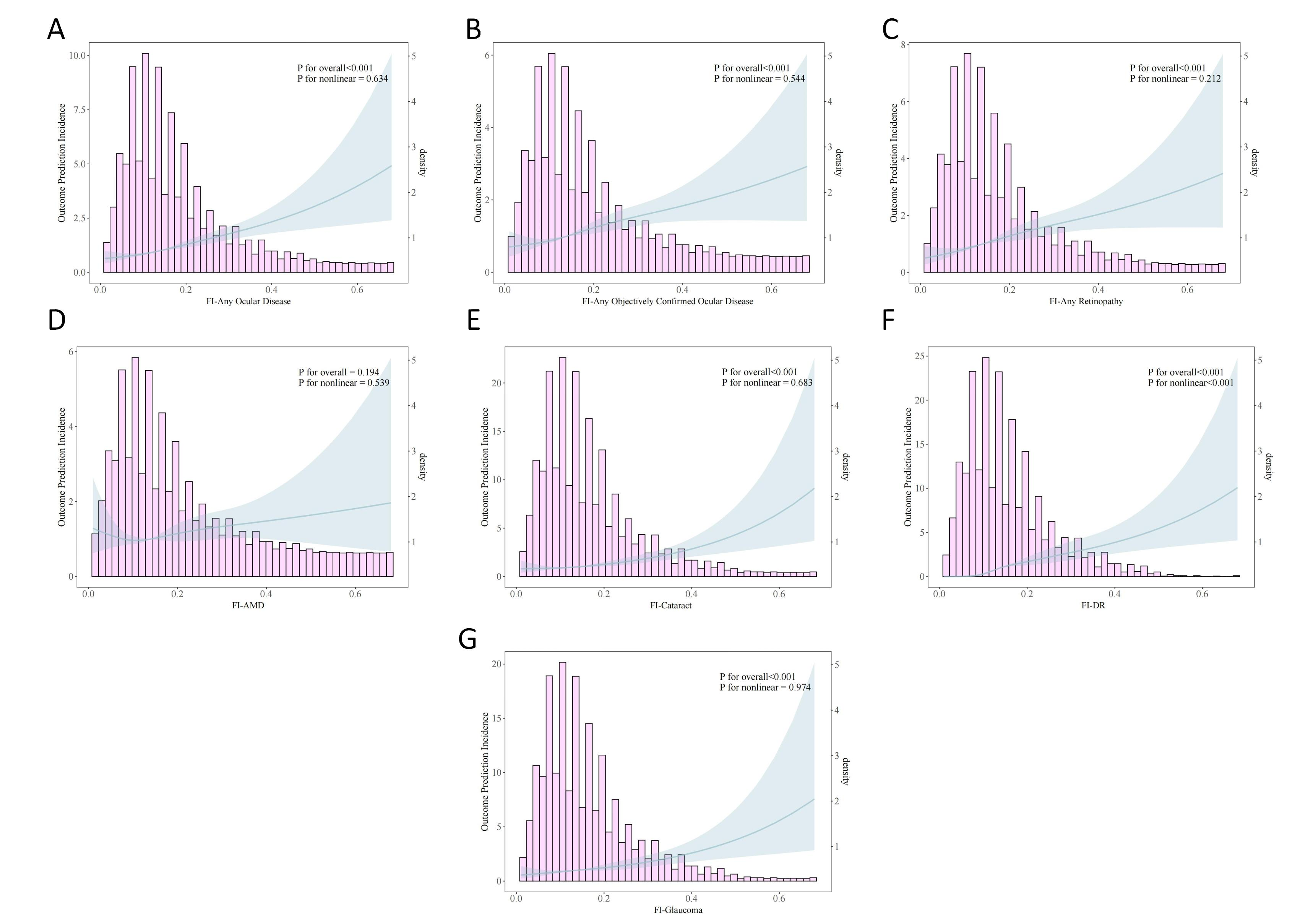


**Figure. S2** Restricted cubic spline plots of the association between FI and major ocular diseases

The results were based on Model 2, which is adjusted for age, sex, ethnicity, educational level, PIR, marital status, smoking, and drinking. Abbreviations: FI, frailty index; AMD, age-related macular degeneration; DR, diabetic retinopathy.


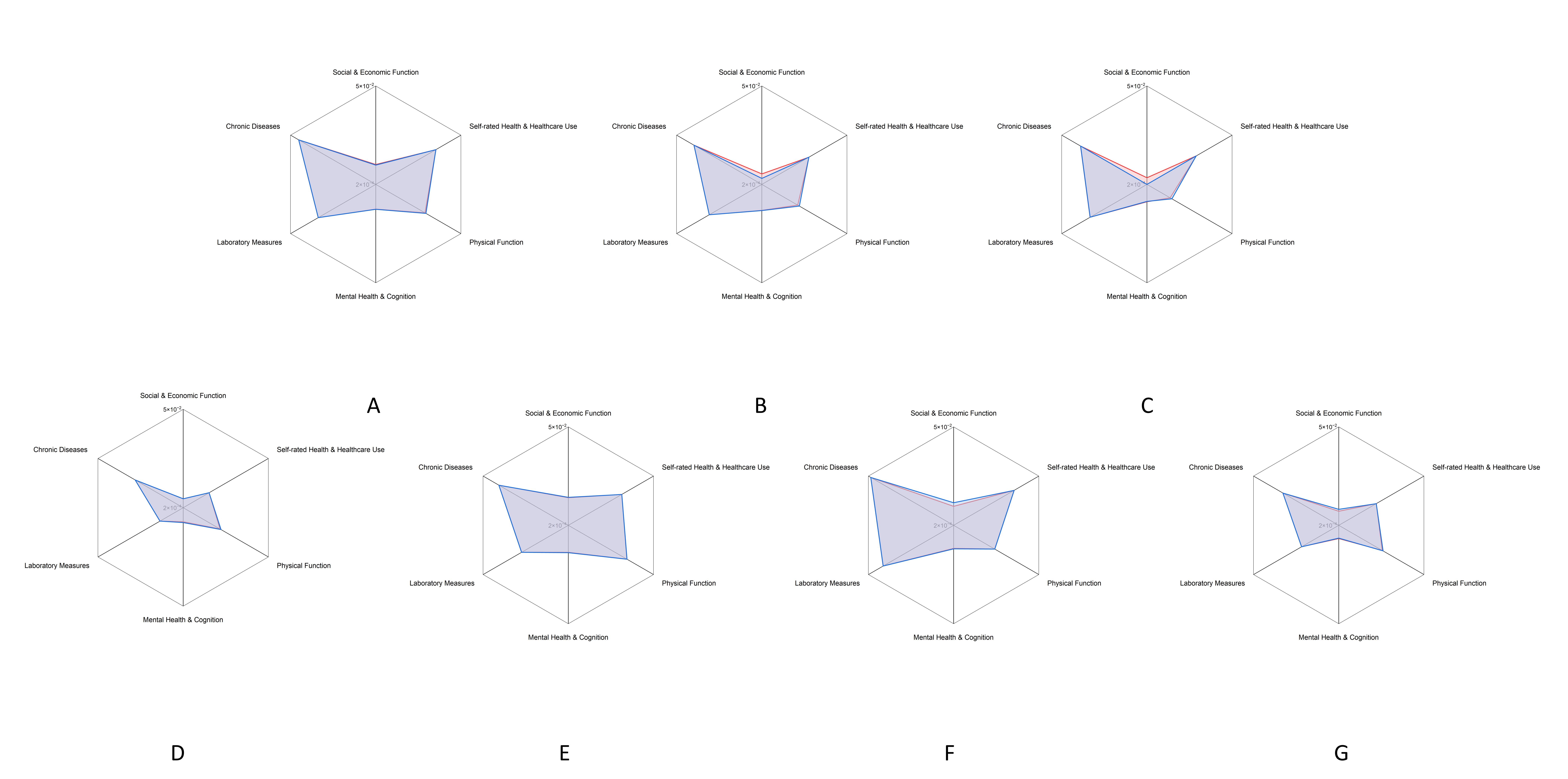


**Figure. S3** Radar plots comparing the absolute contribution values between the main analysis and sensitivity analysis

Panels A–G correspond to “Any ocular disease”, “Any objectively confirmed ocular disease”, “Any retinopathy”, “AMD”, “Cataract”, “Diabetic retinopathy”, and “Glaucoma”. Red lines represent the main analysis (multiple imputation with m = 20 and maxit = 50), and blue lines represent the sensitivity analysis (multiple imputation with m = 5 and maxit = 5). Component analyses are considered exploratory.
